# Human Cysteine Cathepsins Degrade Immunoglobulin G In Vitro in a Predictable Manner

**DOI:** 10.3390/ijms20194843

**Published:** 2019-09-29

**Authors:** Rune Alexander Høglund, Silje Bøen Torsetnes, Andreas Lossius, Bjarne Bogen, E. Jane Homan, Robert Bremel, Trygve Holmøy

**Affiliations:** 1 Department of Neurology, Akershus University Hospital, 1478 Lørenskog, Norway; 2 Clinical Molecular Biology (EpiGen), Medical Division, Akershus University Hospital and University of Oslo, 1478 Lørenskog, Norway; 3 Institute of Clinical Medicine, University of Oslo, 0372 Oslo, Norway; 4 Department of Immunology and Transfusion Medicine, Faculty of Medicine, University of Oslo, 0372 Oslo, Norway; 5 ioGenetics LLC, Madison, WI 53704, USA

**Keywords:** cathepsin, endosome, endolysosome, protease, B cell, antigen presenting cell, bioinformatics, in silico model, protease cleavage prediction

## Abstract

Cysteine cathepsins are critical components of the adaptive immune system involved in the generation of epitopes for presentation on human leukocyte antigen (HLA) molecules and have been implicated in degradation of autoantigens. Immunoglobulin variable regions with somatic mutations and random complementarity region 3 amino acid composition are inherently immunogenic. T cell reactivity towards immunoglobulin variable regions has been investigated in relation to specific diseases, as well as reactivity to therapeutic monoclonal antibodies. Yet, how the immunoglobulins, or the B cell receptors, are processed in endolysosomal compartments of professional antigen presenting cells has not been described in detail. Here we present in silico and in vitro experimental evidence suggesting that cysteine cathepsins S, L and B may have important roles in generating peptides fitting HLA class II molecules, capable of being presented to T cells, from monoclonal antibodies as well as from central nervous system proteins including a well described autoantigen. By combining neural net models with in vitro proteomics experiments, we further suggest how such degradation can be predicted, how it fits with available cellular models, and that it is immunoglobulin heavy chain variable family dependent. These findings are relevant for biotherapeutic drug design as well as to understand disease development. We also suggest how these tools can be improved, including improved machine learning methodology.

## 1. Introduction

The endosomal system of antigen presenting cells (APCs) is home to cysteine cathepsins (B, C, F, H, K, L, O, S, V, W and X), serine cathepsins (A and G), aspartyl cathepsins (D and E), legumain (asparagine endopeptidase, AEP), and gamma-interferon inducible thiol reductase (GILT) [1,2,3]. The expression differs with maturation and activation status of APCs [1,4]. B cells are increasingly investigated as APCs for CD4^+^ T cells, as their APC functions have been connected to disease pathophysiology [1,5,6,7]. Upon activation, the B cell receptor (BCR) and bound antigen are internalized, the antigen is degraded in the endolysosomal system and the resulting fragments of both may bind to major histocompatibility complex (MHC) class II for presentation on the cell surface [1,8,9,10].

Cysteine cathepsins have previously been implied in aging and neurodegenerative disorders and have been detected in microglia, astrocytes, or neurons, in addition to traditional APCs [11]. Further, neuroinflammation is increasingly investigated in what is traditionally considered neurodegenerative disorders [12]. Several central nervous system (CNS) proteins are associated with disease and are either known or potential targets for cysteine cathepsins, including amyloid beta and Tau (Alzheimer’s disease), alpha-synuclein (Parkinson’s disease), and myelin basic protein (MBP, multiple sclerosis) [11].

Monoclonal antibody (mAb) drugs are increasingly being used and developed as therapy for cancer, inflammatory, autoimmune, and other diseases [13,14]. They consist of immunoglobulins (Igs) with constant regions of varying isotypes and allotypes, and variable antigen binding regions of either mouse, human, or humanized origin, which make them inherently immunogenic [15,16]. Development of antibodies towards mAbs is dependent on degradation of Igs by B cells and T cell help [17]. Similar mechanisms have been demonstrated in mice models [18,19].

Observed immunogenicity of therapeutic mAbs could not fully be explained by human leukocyte antigen (HLA)-affinity and T cell epitope predictions alone [20]. Upon internalization of BCR-Ig complexes human GILT allows reduction of disulfide bonds [21,22,23], and endolysosomal proteases likely participate in further degradation of the Igs [24]. In murine bone-marrow derived APCs (non-B cells), cathepsins B and S were important for degrading F(ab’)_2_, after internalization via the FcγR [25]. We have previously described how cysteine cathepsins S, L and B were predicted to cleave human Ig variable regions in specific patterns, and suggested specific roles for these in the degradation of Igs and possibly BCRs allowing presentation of potentially immunogenic fragments on HLA class II [26,27]. Of these cathepsins, S and B are well expressed in B cells, while all three are expressed in monocytes and microglia [1,4,28,29].

While processing and presentation of internalized antigens are frequently investigated, the fate of BCRs upon activation remains poorly described. Still, it has been demonstrated in mice that B cells process and present fragments from their own BCRs on surface MHC class II molecules [10,30,31]. More recently such presentation was found to be extensive in human B cell lymphomas [32,33]. It is likely that cysteine cathepsins degrade both antigen and BCR alike. As Igs and BCRs share common structures [34], understanding degradation of Igs including mAbs could improve our understanding of BCR fragment presentation on HLA class II.

As with other antigens, understanding processing and presentation of Ig requires estimates of processing in the endo-lysosome compartment. Here we present in silico and in vitro experimental validation for cathepsins activity prediction models using CNS proteins including a well described autoantigen (MBP), as well as six therapeutic mAbs. The results suggest that cysteine cathepsins S, L and B effectively degrade both CNS proteins and immunoglobulin G (IgG) in specific and predictable patterns in acidic and reducing conditions simulating endolysosomal compartments.

## 2. Results

### 2.1. Prediction Platform Validation: In Silico Evaluations

Cathepsin peptidases have the ability to cleave many different cleavage site octamers (CSOs) and each enzyme family has activity on substrates that is strongly dependent on the amino acids upstream and downstream of the scissile bond. During the development of the prediction platform it was found that a single general scheme that encompassed all (i.e., 400) different scissile bond dipeptides was not achievable. Thus, an approach was developed wherein each unique P1P1′ scissile bond dipeptide has its own set of neural network (NN) ensembles; each scissile dipeptide in a protein is computed with a neural network ensemble specific for that dipeptide and each cathepsin has several hundred different ensembles. Although the in silico cross-validation of our prediction model platform had previously demonstrated an approximately 90% true positive and 10% false positive rate [35], in an effort to simplify the process here we additionally compared the accuracy to a different machine learning model (support vector machine—SVM) (Supplementary Figure S3), used for binary prediction models such as cleave/no cleavage in the case of cathepsins. In this evaluation, the scissile bond-specific NN ensembles out-performed the SVM in predicting the number of cleavages, indicating that the original NN model is adequately suited for protease cleavage prediction.

### 2.2. Prediction Platform Validation: In Vitro Findings Compared to In Silico Predictions on CNS Proteins

**Figure 1:**
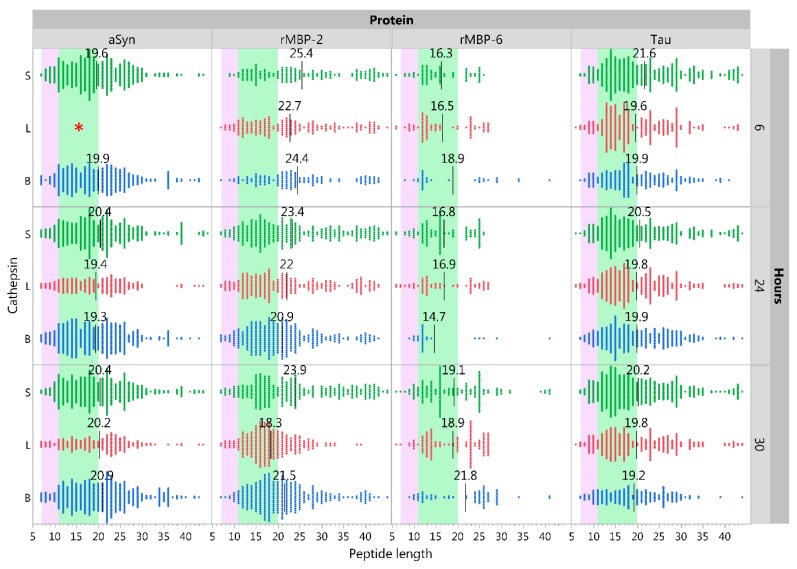
Peptide lengths resulting from in vitro cathepsin digestion of central nervous system proteins. Distribution of peptide lengths after digestion of alpha-synuclein (aSyn), recombinant myelin basic protein (rMBP) isoforms 2 and 6, and tau with either cathepsin B, L, or S at 6, 24, or 30 h at pH 6. Each data point represents one identified peptide at the given time point. Black lines with annotations indicate the mean size of peptides. Purple and green areas indicate peptide sizes fitting HLA class I and II, respectively. * aSyn 6-h sample for cathepsin L was lost due to technical error.

Although training of the NN ensembles employed the best practices available for the task, the size of the training sets is small in comparison to those typically used for large scale artificial intelligence and machine learning. The accuracy of the NN models for full sized proteins had not previously been assessed and the original training set comprised fragments of proteins of partially digested human cells [36]. As different mAbs contain largely similar protein structures, using these alone for validation would cause redundancy in testing. Therefore, we tested the validity of the predictions for full size CNS proteins that may be degraded by cells expressing the cysteine cathepsins (recombinant myelin basic protein [rMBP]-2, rMBP-6, Tau, or α-synuclein), using in vitro experiments at pH 6, as described in the Method section. To evaluate quality of samples, peptides by sample were clustered using Ward’s method (Supplementary Figure S4), showing high similarity between samples with the same protein and cathepsin and no or very few peptides detected in negative controls (30 h incubation). This indicates both lack of impurities or cross-contamination, and sparse spontaneous degradation. The peptide size distribution from different incubation times (Figure 1) indicated substantial cathepsin induced cleavage of the substrates already after 6 h. All cathepsins generated peptides of comparable lengths, ranging from 6 to 45 amino acids, with more than 40% falling into an HLA class II fitting range of 11–20 amino acids after 24 h of cleavage.

**Figure 2:**
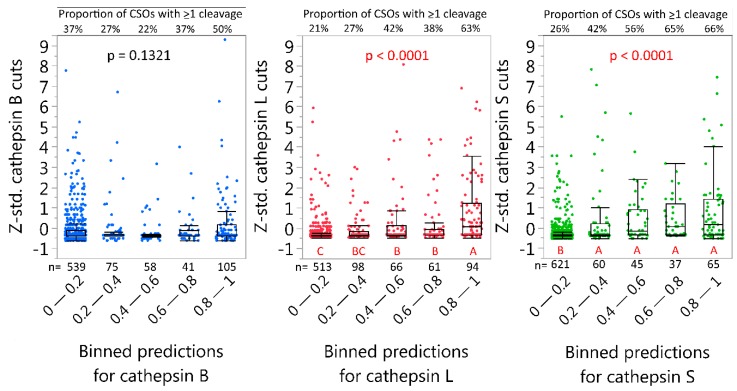
Comparison of predicted and observed cleavage of CNS proteins. All potential cleavage site octamers (CSOs) within alpha-synuclein, recombinant myelin basic protein isoforms 2 and 6, and tau were binned into ranges of 0.2 based on the predicted cleavage probability (X-axis). Intra-protein z-standardized number of observed cuts after 24 h at corresponding CSOs are depicted on the Y-axis. The *p*-values indicate Welch ANOVA significance for cathepsin B/L/S (F(4, 1.53/13.03/12.24)) and differing letters indicate binned groups that have significant difference in mean number of observed cleavages (Tukey–Kramer, HSD). Whiskers are outlier box-plots.

Next, we sought to compare predictions to observed cleavage of the CNS proteins. We quantified and standardized the number of observed cleavages at every CSO after 24 h of incubation with either cathepsin S, L or B using the nano-liquid chromatography mass spectrometry (nLCMS results). The CSOs for all proteins were combined into a single dataset, along with the prediction model cleavage probabilities for the same CSOs. All CSOs were classified by their cleavage probability into grouped ranges (0–0.19, 0.2–0.39, 0.4–0.59, 0.6–0.79, and 0.8–1) and the groups were compared to identify any correlation between cleavage probability and standardized cleavage observations (Figure 2). Of note, CSOs with a low predicted cleavage probability (<0.20) vastly outnumber the other binned groups and reflect the combinatorial effects of the flanking amino acids. The neural net model performed well for cathepsin S and L predictions, as higher predicted probability for cleavage was associated with higher number of cleavages. Also, for over 63% of CSOs with the highest probabilities of cleavage, we observed at least one cleavage. The cathepsin B model underperformed, with a relatively high number of cleavages observed when not predicted (0–0.2 probability). This could possibly be related to its joint endo- and carboxypeptidase capabilities [37]. Such a property will inherently influence the prediction accuracy. This phenomenon is illustrated in Supplementary Figure S5, where the observed number of cleavages for rMBP-2 is plotted by relative maximum distance to a high predicted probability (>0.8) cleavage site. A slight curve-shift to the left could be observed for cathepsin B, but not for L or S, consistent with possible combined endo- and carboxypeptidase activity of cathepsin B.

### 2.3. Cysteine Cathepsins Degrade Immunoglobulins In Vitro

**Figure 3:**
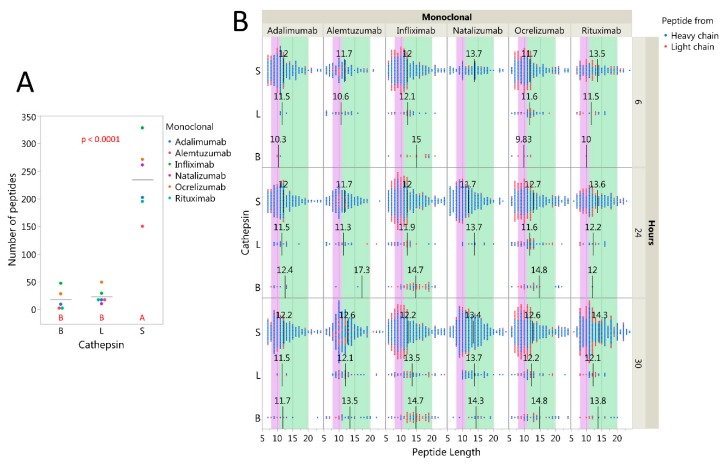
Digestion of monoclonal antibodies at pH 6 by cathepsins S, L and B. Detected peptides after digestion of 1200 nM alemtuzumab, rituximab, natalizumab, or 2400 nM adalimumab, infliximab, or ocrelizumab with either cathepsin B, L, or S at 6, 24, or 30 h at pH 6. (**A**) Cathepsin S yields significantly more detectable peptides than cathepsins L and B at pH 6, after 24 h of incubation. The bars indicate average number of peptides detected. Significance as determined by ANOVA testing and Tukey–Kramer HSD (different red letters indicate significant difference between groups). (**B**) Distribution of peptide lengths (x-axis). Each data point represents one identified peptide at the given time point. Black lines with annotations indicate the mean size of peptides. Purple and green areas indicate peptide sizes fitting HLA class I and II, respectively. The length range is cropped to display 99% of the peptides.

As the NN models performed adequately on peptide cocktails (in silico tests) as well as full sized proteins (in vitro tests), it seemed likely that our previous predicted effects of cathepsins on Igs or BCRs could be relevant [26]. To examine if these cathepsins efficiently degraded Igs, we followed the same procedure as described above for CNS proteins, mixing the mAbs rituximab, natalizumab, alemtuzumab, adalimumab, ocrelizumab, or infliximab individually with each cathepsin at pH 6. Unlike for the CNS proteins, cathepsin S yielded significantly more nLCMS detectable IgG peptides than cathepsins L or B (Figure 3A). The size distributions of IgG peptides were compatible with both HLA class I and II grooves and did not seem to vary much between the different mAbs (Figure 3B). This indicates that single cathepsins can generate IgG fragments for presentation on HLA, and that the cathepsin S, known to be expressed in B cells more than cathepsins L [1], is superior in this function at pH 6.

**Figure 4:**
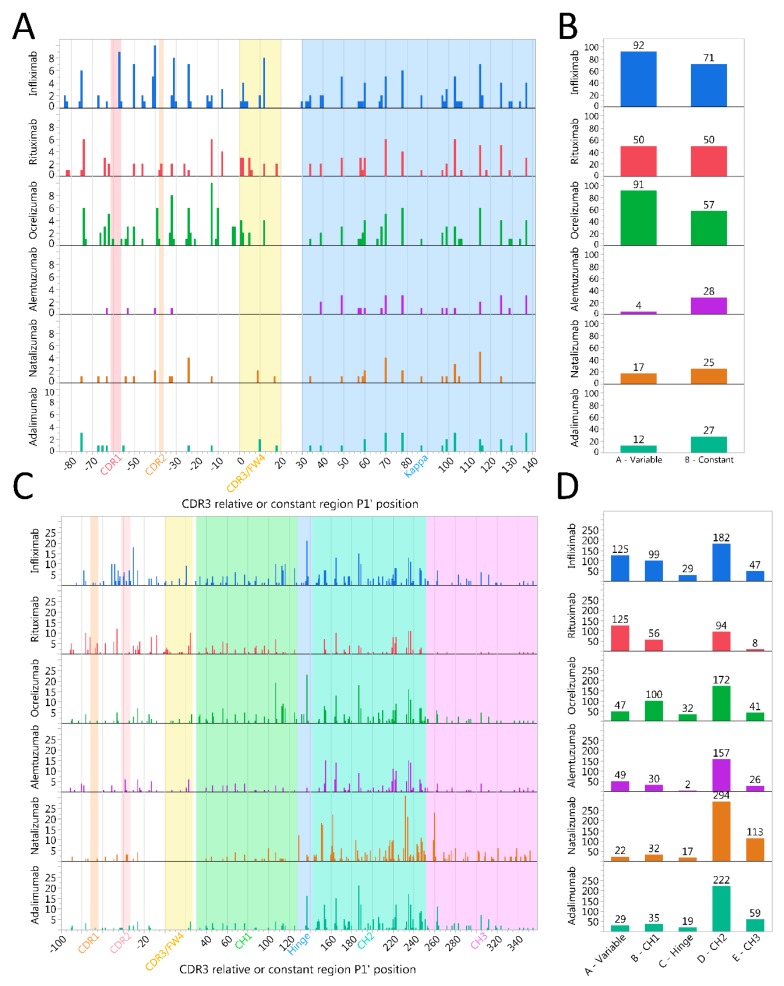
Observed pattern of cathepsin S cuts in monoclonal antibodies. The monoclonal antibodies adalimumab, alemtuzumab, infliximab, natalizumab, ocrelizumab, and infliximab were incubated with cathepsin s for 24 h at pH 6. Non-standardized number of observed cuts in light (**A**/**B**) and heavy chains (**C**/**D**) identified by nano-liquid chromatography mass spectrometry. Cuts are presented by their location in sequence (**A**/**C**) or summarized by region (**B**/**D**). For alignment purposes, the variable region position is assigned by the relative position of P1′ in the cleavage site octamer to the cysteine (0) of CDR3. The constant regions are aligned to start at position 30.

As the size distribution of IgG peptides were compatible with HLA presentation, we went on to investigate from which regions these peptides were derived, focusing mainly on cathepsin S. The primary protein structures of the heavy and light chains of all six mAbs (Table S1) were utilized to align the identified peptides to the corresponding amino- and carboxy-end cleavage locations. Figure 4A,C display a relatively fixed pattern of degradation for constant regions of both heavy and light chains. A small cleavage location shift was observed for natalizumab heavy chain, due to the inherent sequence difference between IgG4 and IgG1. Interestingly, the heavy constant 2 regions seemed to be most sensitive to cleavage across the mAbs. Thus, the cathepsins demonstrated a capability of cleaving a variety of CSOs consistently across several mAbs. Cleavages observed for the variable regions contrasts this, as patterns differed between the mAbs (Figure 4A,C). A notable difference was the higher number of observed cleavages and cleavage positions in heavy chains for the chimeric infliximab and rituximab compared to the other mAbs, which carry humanized or human variable regions (Figure 4C,D).

### 2.4. Neural Net Prediction Accuracy for Immunoglobulin Cathepsin Cleavage

**Figure 5:**
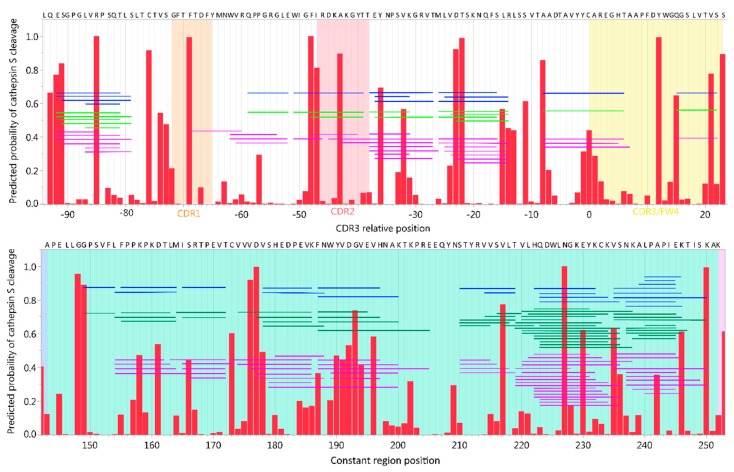
Detected peptides overlap with predicted cleavage sites. Predicted cleavage probability (x-axis) by cathepsin S in variable (upper panel) and constant heavy 2 (CH2) (lower panel) region of alemtuzumab. The vertical bars indicate the predicted position of P1′ of a P1-P1′ cleavage bond, and thus the first amino acid after a cut. Horizontal bars each indicate unique peptides detected starting at a P1′ and ending at a P1, as identified by nLCMS after 6 (blue), 24 (green), and 30 (purple) hours.

Based on the above results cleavage within the variable region is likely important for the immunogenicity of therapeutic mAbs. As the model can be used to individually assess the likelihood for such cleavage, we assessed the peptide distribution qualitatively, compared to predicted cleavage sites for alemtuzumab heavy chain variable and constant region 2 (Figure 5). Notably, many peptides seem to be derived from longer fragments and either start or end at a predicted cleavage site, but not necessarily a site with high probability of cleavage (>0.8). A larger pool of unique peptides was detected after 30 h than after 6 h (Figure 3B and Figure 5).

**Figure 6:**
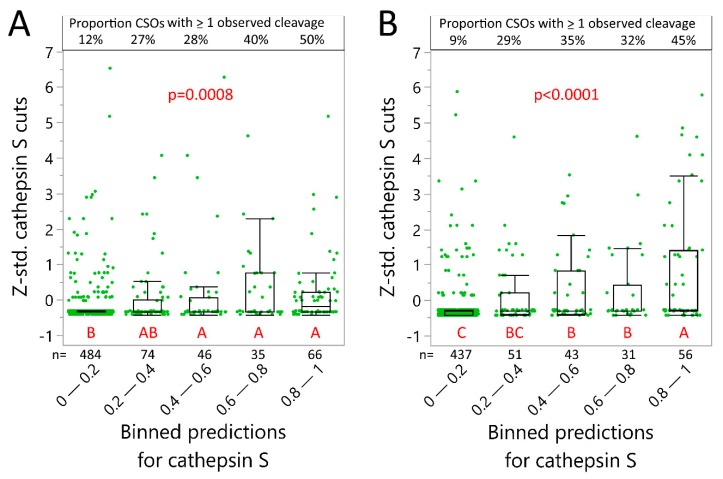
Evaluation of cleavage accuracy for monoclonal antibody variable regions. Cleavage probability by cathepsin S for all possible cleavage site octamers (CSOs) within (**A**) heavy and (**B**) light chain variable regions of rituximab, infliximab, ocrelizumab, natalizumab, alemtuzumab, and adalimumab were binned into ranges of 0.2 (X-axis). Intra-chain z-standardized number of observed cuts after 24 h at pH 6 are depicted on the Y-axis. *p*-values indicate Welch ANOVA significance (F(4, 5.16/9.05) for heavy and light respectively), and differing red letters indicate significant differences between groups (Tukey–Kramer, HSD). Whiskers are outlier box-plots.

As with the CNS proteins, we further tested statistically the predictive models’ accuracy for Ig variable region cleavage at pH 6 in a binned analysis. Cathepsin S predictions performed well, with high cleavage probability being associated with higher number of cleavages but were not as accurate as for the CNS proteins (Figure 6). For instance, only 45–50% of high probability cleavage sites had at least one cleavage observation. In addition, peptides found from the shorter IgG light chain seemingly fit better with predictions than heavy chain. This and the patterns shown in Figure 5 indicated that longer fragments resulting from incomplete cleavage, with lengths exceeding nLCMS method limitation, potentially remained undetected. Not surprisingly, the accuracy for cathepsins B and L was not as good as with cathepsin S, given fewer peptides on which to base the analysis (Supplementary Figure S6).

### 2.5. Influence of pH on Cathepsin Activity

**Figure 7:**
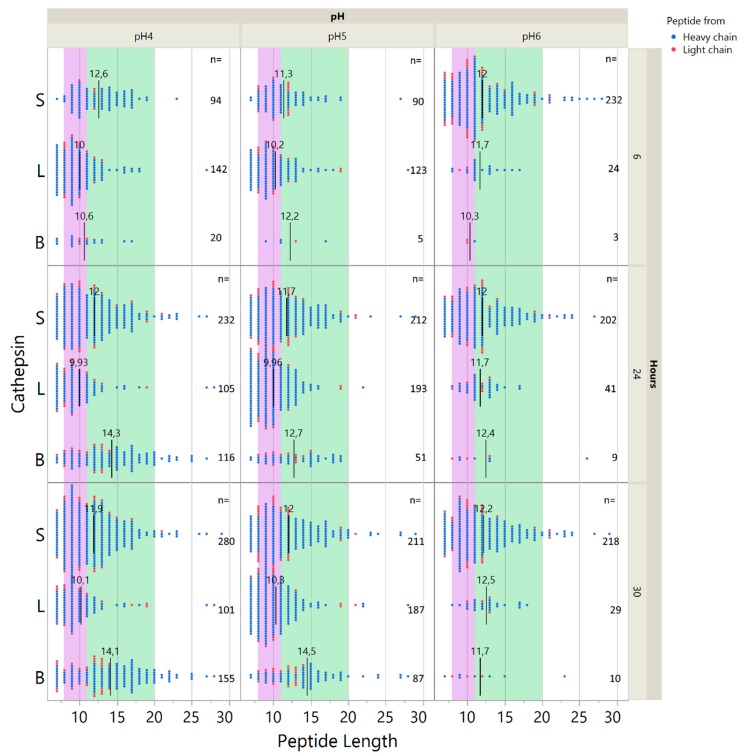
Adalimumab digestion by cathepsin S, L and B at pH 4, 5, and 6. Distribution of peptide lengths after digestion of 2400 nM adalimumab with either cathepsin B, L, S at 6, 24, or 30 h at pH 4, 5, or 6. Each data point represents one identified peptide at the given time point. Black lines with annotations indicate the mean size of peptides. Purple and green areas indicate peptide sizes fitting HLA class I and II, respectively. (Note: For pH 6, the data for cathepsins S and B are the same as in Figure 3B).

The pH optimum for cathepsins differs. Moreover, DTT reducing efficiency wanes at low pH [38], offering less reduction of IgG disulfide bonds that also could influence degradation patterns. We therefore further tested digestion by cathepsins S, L and B at pH 4 and 5 in addition to pH 6 (using only 1:100 enzyme to substrate ratios). Cathepsins L and B generated more peptides at lower pH, while pH had little influence on peptide yield for cathepsin S (Figure 7). Similar results were obtained for infliximab (Figure S8). The cathepsins also showed a relatively conserved cleavage pattern across multiple pHs for adalimumab, best illustrated by cathepsin S (Supplementary Figure S7) due to its preserved activity at pH 6. However, cathepsin L and B also display high levels of similarities when comparing pH 4 to pH 5 results (Supplementary Figure S7). As predictive models were built using datasets generated at pH 6, we did not test prediction accuracy at pH 4 and 5.

To assess whether DTT activity in fact was reduced at low pH, we performed sodium dodecyl sulfate polyacrylamide gel electrophoresis (SDS-PAGE) assays to assess residual mAb multimer structures. Negative samples were run with both reducing and non-reducing running buffers to account for reduction occurring in cleavage assay as well as the SDS-PAGE assay. It is evident that DTT activity was far more potent at pH 6 than pH 4, as several larger structures remained intact at pH 4 (Figure S9A). These likely reflected various combinations of heavy- and light chains sized 75 kDa (heavy + light), 100 kDa (2× heavy), 125 kDa (2× heavy + light), and 150 kDa (full IgG). Another observation was that bands around 50 kDa (heavy) and 25 kDa (light) were still abundant at pH 6, indicating incomplete degradation of these even in presence of reducing conditions, both for cathepsin incubated samples and negative control samples. The loss of multimeric structures was time dependent, as demonstrated for ocrelizumab at pH 5 in Supplementary Figure S9B. Apart from cathepsin L at pH 4, only small differences were observed between cathepsin samples and negative controls for all pHs, implying that a considerable amount of heavy and light chains remained intact even after cathepsins processing.

The cathepsins require reduction by e.g., DTT for activation but are also capable of auto-catalytic activation at acidic pH [37,39,40]. We observed that cathepsin activity was present despite loss of DTT efficiency, which indicated that the cathepsins most likely were auto-catalytically activated at acidic pH. Also, results indicate that cathepsins did not fully degrade the IgGs, which is compatible with a limited proteolytic activity for optimal generation of epitopes [41].

### 2.6. Immunoglobulin Heavy Variable Gene Family Determines Different Cleavage Patterns

**Figure 8:**
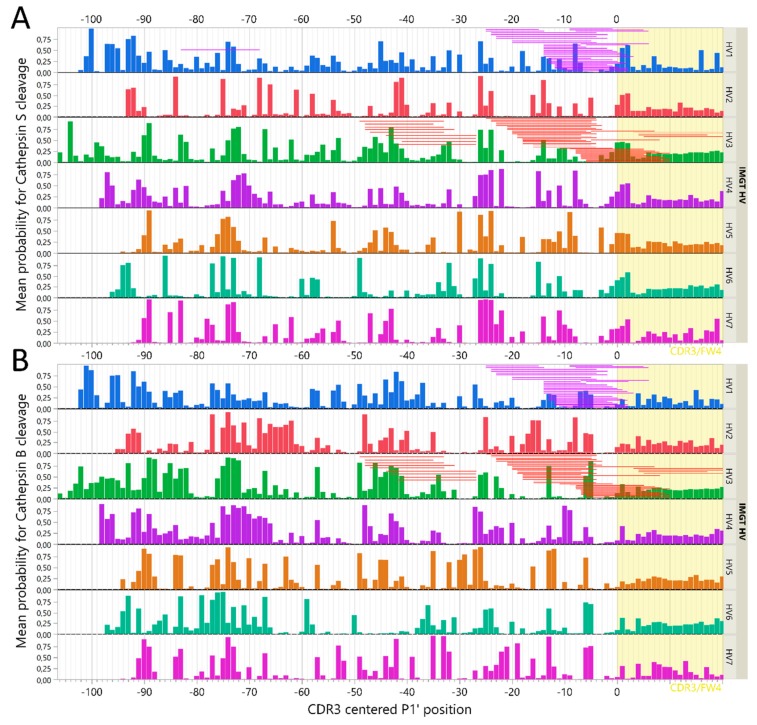
Predicted cleavage patterns of IGHV families using GenBank IGHV set. Approximately 16,000 curated IGHV sequences were divided by their V-family and analyzed with the cathepsin cleavage models. All possible cleavage site octamers (CSO) for each IGHV were aligned according the relative position of P1′ to the CDR3 (yellow) region cysteine (x-axis). Mean predicted probability for CSO cleavage at each position by cathepsin S (**A**) or cathepsin B (**B**) is shown on the y-axis. Superimposed are aligned IGHV peptides described by Khodadoust et al. (31), eluted from HLA class II of mantle cell lymphoma: MCL065 (purple) and MCL052 (red).

Differences in amino acids patterns between the different IGHV families could likewise modulate the cathepsin cleavage patterns and thus be critical for immunogenicity of therapeutic mAbs. Previous data indicated that the immunoglobulin heavy variable (IGHV) family may dictate differences in degradation [26], and our findings here confirmed that such differences may be predicted to some extent. Thus, we sought to identify differences and/or similarities by using a previously assembled Ig variable region library [42], and plotting mean predicted cleavage probabilities for all CSOs using the C-terminal cysteine of CDR3 as an alignment to coordinate the relative position of P1′ (Figure 8). The mean probability of cleavages for cathepsin S clearly demonstrated different patterns of degradations by IGHV family, although some features were preserved. Notably, at CDR3 relative position −26, there was a preserved high probability for a cathepsin S cleavage site across all IGHV families, that was also consistently identified for all mAbs assessed with cathepsin S in vitro (Figure 4 and Figure 8). In addition, a less pronounced but consistent increase in probability for cathepsin S cleavage across IGHV families was observed at the beginning of CDR3 (Figure 8). IGHV 3 had the lowest predicted cathepsin S probabilities for cleavage in the framework 3 region, consistent with our previous findings [26].

To investigate the validity of these assessments, we further compared the cathepsin B and S cleavage prediction data with those reported from IGHV-derived peptides eluted from HLA class II on lymphoma cells from two patients with mantle cell lymphoma [33]. As the full IGHV sequences of these clones were not available, we assembled the most complete IGHV sequence possible from each cell line (MCL052 and MCL065) and aligned them using the International Immunogenetics Information System (IMGT) database standards and assigned the assembled sequences to an IGHV family [43]. Then, the peptides were aligned according to the CDR3-relative position and compared to the observed cleavage pattern with predicted output of GenBank sequences as well as the mAb cleavage assays (Figure 8). Notably, many identified cleavage sites from the lymphoma IGHV peptides could be explained by either cathepsin B or S activity. For instance, cleavage around CDR3-relative position −26 for MCL052 (IGHV3) is compatible with cathepsin S protease activity, as it is evident from both the GenBank set and observed cuts in IGHV3-carrying mAbs (ocrelizumab and adalimumab). Likewise, consistent cleavage around position −5 can be explained by cathepsin B activity for IGHV3. Notably, several predicted cleavage sites confirmed by our in vitro studies were not identified in these peptides, possibly indicating a protective role of HLA class II binding. Compatible with this, IGHV 15-mers starting around CDR-3 relative positions −40, −20, and −5, as well as within the CDR3, was previously predicted to have high affinity for HLA-DR molecules [26,42].

## 3. Discussion

We hypothesized that CNS proteins and Ig variable regions are degraded in predictable patterns by cysteine cathepsins S, L and B in endolysosomal compartments of APCs [26]. Here, we have demonstrated such degradation patterns in vitro, showing how these cathepsins all degrade CNS proteins and IgGs into peptides sized to fit in HLA class II under conditions resembling the endolysosomal compartments. Further, we have validated in silico neural net models that can predict the pattern of such proteolysis.

The endolysosomal compartments are acidic and reducing [3], allowing proteases to degrade most foreign and self-proteins. Cathepsin L and S have both been attributed key importance in degrading class II-associated invariant chain peptide (CLIP) and preparing MHC class II for antigen binding, as well as antigen processing in general [1,2]. Several cathepsins are found in CNS cells [28], and cathepsin S and B in particular have suggested roles in neurodegenerative diseases [44]. It has been shown that cathepsin S has an important role in degradation of MBP [45], and we identified several peptides investigated for their potential immunogenicity (MBP_13–32_, MBP_131–155_, and MBP_146–170_) [46], or associated cleavage sites, after cleavage with cathepsins L or S. Another variant, MBP_83–99,_ was both predicted and found to be destroyed by cathepsins S and L, as has also been described previously [45].

With heterogeneous degradation cleavage patterns [36] and presence in antigen presenting cells [1], a potential role for cathepsins S, L and B in degrading diverse Igs seemed likely. In this study, we confirmed that these cathepsins cause IgGs to be degraded in a pattern determined by their structure, as is evident from a fixed degradation pattern of constant region, and differing patterns in the variable regions.

Therapeutic mAbs are generally designed to minimize immunogenicity [47], yet anti-drug antibodies remain problematic. IgG antibodies make up the majority of anti-drug antibodies [48], and generation of such antibodies requires T cell help [17,19]. Due to the diversity of variable regions of heavy and light chains, we assume that the immunogenic T cell epitopes are derived from the variable regions, and several tools exist to make predictions to find them [49]. However, as data on Ig processing has been lacking, assumptions on processing are frequently absent in these tools. Here, we showed that cathepsins expressed by B cells efficiently generate epitopes from IGHV regions. Interestingly, chimeric antibody heavy chain variable regions were particularly prone to degradation, possibly contributing to their higher immunogenicity [20]. Parallel to this, it was shown that peptides introduced into human heavy constant 2 regions were more effectively presented on MHC II in mice than peptides inserted into the other domains [50], consistent with the observed higher number of cleavages within this region (Figure 4C).

We and others have previously suggested that mutations in the IGHV region could break T-cell tolerance towards B cell receptors in vivo, leading to autoimmune disease [27,51,52,53]. Any small change, be it introduced by mutation or by design, could influence cathepsin cleavage patterns, and thus which IGHV peptides are presented. We have further attempted to model likelihood of such T-cell responsiveness to IGHV variable regions, using a combination of HLA class II affinity and cleavage by either cathepsin S, L or B [26]. However, the results of this study were based on in vitro experiments, that are not necessarily directly comparable to full-scale intracellular processing, and do not encompass the full complexity of the endolysosomal compartments. The intracellular machinery resulting in HLA class II presentation is intricate, involving a suitable cell activation state, endosomal environment, multiple cathepsins, GILT, HLA class II, and HLA-DM [54]. Protection from digestion by HLA class II binding may be particularly relevant. Nevertheless, several studies have published epitope libraries (www.iedb.org, [55]) demonstrating that peptides from Igs and/or BCRs are presented frequently on different APC’s HLA class II molecules [56,57,58,59], and a few also performed IGHV sequencing to achieve an optimal search database [32,33]. Interestingly, IGHV peptides derived from dendritic cells loaded with therapeutic intravenous Igs [57] share similarities with IGHV peptides derived from self BCR in lymphomas [32,33], suggesting a similar mechanism of degradation.

It has been suggested that predicted cathepsin cleavage patterns did not explain HLA class II eluted IGHV peptides from the lymphomas [32]. This assessment may not have accounted for differential degradation of the IGHV families, nor the predicted high affinity for HLA-DR molecules of peptides in the framework 3 region [26,42]. We found that several HLA class II eluted IGHV peptides could be explained by either cathepsin B or S (Figure 8). Likewise, another group eluted HLA class II bound peptides from DCs incubated with infliximab or rituximab, and found several peptides compatible with both our predicted pattern and our observed peptides after cleavage with individual cathepsins [60]. Cleavage sites not explained by cathepsins described here, are likely the result of other endosomal proteases, including cathepsin H, as demonstrated for other substrates in more complex in vitro models [54], or legumain cleaving aspartic or asparagine bonds [61,62].

Based on the nLCMS results alone, one could presume that the IgGs were completely degraded by cathepsins, particularly at lower pH values. Yet, SDS-PAGE experiments unveiled a significant amount of heavy and light chains with relatively high molecular weights remaining after in vitro cathepsin processing. Additionally, cathepsin degradation may potentially have rendered some larger fragments that were not detected by gel analysis, due to differences in size and/or cleavage position. Even with the high sensitivity of a mass spectrometer, it is not possible to detect every cleavage site due to detection restrictions of the nLCMS instrument (typically 6–40 amino acid peptides). We also assume that identification of degradation close to a free carboxyl- or amino-end will be somewhat overestimated compared to that in the middle of large structures. This will skew the nLCMS output, and potentially explain a poorer prediction accuracy for the heavy chains. In complete endolysosomal systems of APCs, these restrictions may not apply, as different cathepsins likely work in tandem under reducing and increasingly acidic conditions to ensure proper degradation. In vitro models including multiple cathepsins [54], or unbiased HLA-elution assays accounting for both processing and HLA binding [33,63], can generate training sets further improving cleavage accuracy prediction of neural net models.

Cathepsin-generated epitopes are likely important for eliciting anti-drug antibodies, and the knowledge of these mechanisms is therefore important in the design of future therapeutic mAbs. Specific insight into B cell expressed cathepsin degradation of IgGs, as shown here, can supplement traditional epitope-mapping tools.

## 4. Methods

### 4.1. Cathepsin Cleavage Predictions

It is common practice to consider the amino acid contacts in a CSO, comprising ± 4 amino acids from the scissile bond, as the peptide contact region of a peptidase [64]. Cleavage occurs between amino acids 4 and 5 of the CSO. We have previously described the conversion of amino acid sequences into matrices of principal components of the physical properties of the amino acids as the input layer of neural networks [65,66]. For this study prediction of cleavage probability for cathepsin S, L and B were done with neural network models as described previously [26,35], trained using proteome derived-peptide library datasets from Biniossek et al. [36]. The method used was analogous to one used to predict peptide affinities for HLA class I and II [65,66]. In brief, neural net ensembles for each cathepsin were trained using principal components of amino acid physical properties of the CSO (Figures S1 and S2) to predict the cleavage probability of a peptide bond P1-P1′ of any P4P3P2P1-P1′P2′P3′P4′ octamer. Amino acid sequences were converted to 3-row matrices using the first three principal components that comprises approximately 90% of the variance in a range of different physical properties commonly used in structural biology [66]. The output of the neural networks ranged between 0 (low-) and 1 (high) probability for cleavage. Pseudo code for the training process is given in Figure S2 and derivation of the activation functions of the neural networks were done with the “Neural” platform of JMP^®^ (SAS Institute, Cary, NC, USA). As the input is the primary amino acid sequence of proteins, once derived, the activation functions can be used to make predictions of any protein divided into sequential potential CSO. More details can be found in the supplemental section of an earlier publication [35], but are similar to those in common use in artificial intelligence modeling.

Predictions for cathepsin S, L and B cleavage were computed for every potential CSO in all substrates described below, as well as for 16.000 IGHV sequences previously curated from GenBank [42]. For IGHV sequences, family was assigned according to IMGT [43], and CSO P1′ positions were indexed and aligned by their relative position to the cysteine marking the beginning of CDR3 (position 0).

### 4.2. Cathepsins and Substrates of the In Vitro Cleavage Assays

In this study we used recombinant human cathepsin S (UniprotKB P25774), L (UniprotKB P07711), and B (UniprotKB P07858) from R&D Systems (Biotechne, Minneapolis, MN, USA). The two types of substrates in this study were proteins derived from the central nervous system and therapeutic mAbs. The CNS proteins were rMBP isoform 2 (Uniprot KB P02686-2) and a variant of isoform 6 (P02686-6) (LSBio, Seattle, WA, USA); recombinant alpha synuclein (α-synuclein) isoform 1 (P37840-1, rPeptide, Watkinsville, GA, USA), and microtubule-associated protein tau (Tau) isoform Tau-F (P10636-8, rPeptide, Watkinsville, GA, USA). The therapeutic mAbs were alemtuzumab (Lemtrada^®^, Genzyme, Cambridge, MA, USA), natalizumab (Tysabri^®^, Biogen, Cambridge, MA, USA), rituximab (Rixathon^®^, Sandoz, Holzkirchen, Germany), ocrelizumab (Ocrevus^®^, Roche, Basel, Switzerland), adalimumab (Humira^®^, Abbvie, North Chicago, IL, USA), and infliximab (Inflectra^®^, Pfizer, New York, NY, USA).

The cathepsins were mixed with substrate at ratios of 1:100 or 1:300 (*w*/*w*) in 50 mM sodium phosphate, 200 mM NaCl, 5 mM EDTA, and 4 mM dithiothreitol (DTT) at pH 4, 5, or 6. Substrate concentrations were 1200 or 2400 nM, to extensively exceed the mass spectrometry detection limit. Samples were incubated at 37 °C, on a shaker plate at 300 rpm for up to 30 h. Aliquots were incubated for 6, 24, or 30 h, and immediately frozen at −20 °C to stop catabolic activity. For each substrate, a negative control without cathepsin was run parallel to the experiments.

### 4.3. Nano Liquid Chromatography Mass Spectrometry and Related Software for Data Processing

All machines, equipment and software used for nLCMS were from Thermo Fisher Scientific (Waltham, MA, USA) unless otherwise stated.

The instrument performing nLC separation was a nano EasyLC1000, equipped with Accucore 150-C4 pre- and analytical columns (0.3 × 5 mm and 0.075 × 150 mm) used in a vented 2-column setup. Mobile phases (MPs) were 0.1% formic acid in H_2_O (MPA) and 0.1% formic acid in acetonitrile (MPB). Loading solution was also MPA and a volume of 5 uL of sample was injected at the flowrate 3 μL/min for every analysis. The mass spectrometer (MS) acquisition was turned on after injection and during acquisition the analytical flow rate was constant at 400 nL/min, being initially isocratic with 1% MPB for 1 min, before MPB was ramped up from 1% to 50% in 10 min and then from 50% to 70% in 2 min.

Mass spectrometry was performed with a QExactive Orbitrap with a heated electrospray ionization source operated at +2 kV. Data was acquired in a data-dependent manner by the following parameters: resolution of 70,000 in MS and 17500 in MS/MS, scan range from 350–1350 *m*/*z*, AGC target of 1e6 for MS, and 1e6 for MS/MS, top 7 selected for fragmentation, dynamic exclusion of 5 s, and exclusion of unknown charge.

Method setup and data acquisition was controlled by the Xcalibur^TM^ software (version 2.2), while data processing and identification of peptides was performed using MaxQuant version 1.6.1.0 with the built in Andromeda search engine (freeware available at maxquant.org) [67]. Peptide false discovery rate was set to 0.01, and a mass tolerance of 5 ppm and 25 ppm was used in MS and MS/MS, respectively. Cleavage specificity was set to unspecific and methionine oxidation, N-terminal acetylation and asparagine deamidation were used as variable modifications, though no modified variants were detected.

The full sequences for substrates were acquired from the Uniprot database [68], and from the IMGT 2D/3Dstructure mAb-database or patent filings [69]. In some cases, the constant regions were imputed from existing literature [16]. The mAb sequences used are listed in Supplementary Table S1.

### 4.4. SDS-PAGE

SDS-PAGE was run under reducing or non-reducing conditions with one µg of select samples to assess residual IgG fragments with sizes exceeding the optimal nLCMS detection range (about >40 amino acid length). We utilized 4–20% Criterion^TM^ TGX (Bio-Rad, Hercules, CA, USA) gels and Laemmli sample buffer (Bio-Rad, Hercules, CA, USA) with or without 50mM 1,4-dithiothreitol (DTT, Sigma-Aldrich, St. Louis, MO, USA) prepared according to the manufacturer’s instructions. Gels were stained with Coomassie blue G-250 (Bio-Rad, Hercules, CA, USA) and photographed using ChemiDoc^TM^ XRS+ with Image Lab™ software version 6.0.0 (Bio-Rad, Hercules, CA, USA).

### 4.5. Statistics

All statistical analyses and graphics were performed in JMP^®^ Pro 14.1 (SAS Institute, Cary, NC, USA). For statistical testing of number of cleavages across proteins, intra-protein z-standardization of observed cleavage frequencies was used to improve comparability across substrates of differing lengths and/or concentrations. The accuracy of the models was only evaluated statistically for conditions emulating the conditions of the training sets (35). Unless otherwise stated, figures depict the cleavage position as the P1′ of the CSO, indicating the position of the first amino acid after cleavage. For graphic output purposes the position of P1′ was assigned relative to the cysteine at the start of CDR3 for variable regions and constant regions were aligned to start at position 30.

## 5. Conclusions

Using mass spectrometry proteomics techniques, we have demonstrated that NN ensembles derived using the principal components of physical properties of amino acids flanking the scissile bond, can predict in vitro proteolysis of both CNS proteins and mAbs by cathepsins S, L and B. While the constant regions of Igs follow a highly reproducible pattern of degradation, variable regions display differing patterns that are related to their IGHV family structure. This knowledge may be essential for understanding immune responses against both endogenous Igs and BCRs as well as therapeutic mAbs. As NN training is an ongoing process the CSO peptides in this study will enable re-training of the NN, improving their accuracy. These results further suggest that directed efforts towards expanding the knowledge base regarding the specificity and expression patterns of peptidases involved in antigen presentation is warranted.

## Supplementary Materials

Supplementary materials can be found at https://www.mdpi.com/1422-0067/20/19/4843/s1. Proteomics data from cathepsin cleavage assays can be found at: https://doi.org/10.6084/m9.figshare.9777725.

## Abbreviations

APC	antigen presenting cell
BCR	B cell receptor
CDR3	complementarity determining region 3
CNS	Central nervous system
CSO	cleavage site octamer
HLA	human leukocyte antigen
Ig	immunoglobulin
IgG	immunoglobulin G
IGHV	immunoglobulin heavy variable
mAb	monoclonal antibody
MHC	major histocompatibility complex
NN	neural network
nLCMS	nano liquid chromatography mass spectrometry
rMBP	recombinant myelin basic protein
SDS-PAGE	sodium dodecyl sulphate-polyacrylamide gel electrophoresis
SVM	support vector machine
