# Supplementary material for: Human Cysteine Cathepsins Degrade Immunoglobulin G In Vitro in a Predictable Manner
Source: Int J Mol Sci. 2019 Sep 29;20(19):4843. doi: 10.3390/ijms20194843 (PMC6801702; doi:10.3390/ijms20194843)
Supplement: Supplementary file 1 [file ijms-20-04843-s001.pdf]

Supplementary materials

Human cysteine cathepsins degrade immunoglobulin G *in vitro* in a predictable manner

Rune A. Høglund[1,2,3†], Silje Bøen Torsetnes[1,2†], Andreas Lossius[1,2,4], Bjarne Bogen[4], E. Jane Homan[5], Robert Bremel[5], Trygve Holmøy[1,3]

[1]Department of Neurology, Akershus University Hospital, Norway

[2]Clinical Molecular Biology (EpiGen), Medical Division, Akershus University Hospital and University of Oslo, Lørenskog, Norway

[3]Institute of Clinical Medicine, University of Oslo, Norway

[4]Department of Immunology and Transfusion Medicine, Faculty of Medicine, University of Oslo, Norway

[5]ioGenetics LLC, Madison, Wisconsin, USA

†These authors contributed equally

Table S1 Included immunoglobulins for the study

general identifier[1]	monoclonal	Isotype	Chain	Allotype	V family[2]	Full sequence (VH-CH)	Length
7602	infliximab	IgG1k	L†	Km3	5	DILLTQSPAILSVSPGERVSFSCRASQFVGSSIHWYQQRTNGSPRLLIKYASESMSGIPSRFSGSGSGTDFTLSINTVESEDIADYYCQQSHSWPFTFGSGTNLEVKRTVAAPSVFIFPPSDEQLKSGTASVVCLLNNFYPREAKVQWKVDNALQSGNSQESVTEQDSKDSTYSLSSTLTLSKADYEKHKVYACEVTHQGLSSPVTKSFNRGEC	214
			H†	G1m17	6/3	EVKLEESGGGLVQPGGSMKLSCVASGFIFSNHWMNWVRQSPEKGLEWVAEIRSKSINSATHYAESVKGRFTISRDDSKSAVYLQMTDLRTEDTGVYYCSRNYYGSTYDYWGQGTTLTVSASTKGPSVFPLAPSSKSTSGGTAALGCLVKDYFPEPVTVSWNSGALTSGVHTFPAVLQSSGLYSLSSVVTVPSSSLGTQTYICNVNHKPSNTKVDKKVEPKSCDKTHTCPPCPAPELLGGPSVFLFPPKPKDTLMISRTPEVTCVVVDVSHEDPEVKFNWYVDGVEVHNAKTKPREEQYNSTYRVVSVLTVLHQDWLNGKEYKCKVSNKALPAPIEKTISKAKGQPREPQVYTLPPSREEMTKNQVSLTCLVKGFYPSDIAVEWESNGQPENNYKTTPPVLDSDGSFFLYSKLTVDKSRWQQGNVFSCSVMHEALHNHYTQKSLSLSPGK	449
7609	rituximab	IgG1k	L	Km3	4	QIVLSQSPAILSASPGEKVTMTCRASSSVSYIHWFQQKPGSSPKPWIYATSNLASGVPVRFSGSGSGTSYSLTISRVEAEDAATYYCQQWTSNPPTFGGGTKLEIKRTVAAPSVFIFPPSDEQLKSGTASVVCLLNNFYPREAKVQWKVDNALQSGNSQESVTEQDSKDSTYSLSSTLTLSKADYEKHKVYACEVTHQGLSSPVTKSFNRGEC	213
			H	G1m17,1	1/1	QVQLQQPGAELVKPGASVKMSCKASGYTFTSYNMHWVKQTPGRGLEWIGAIYPGNGDTSYNQKFKGKATLTADKSSSTAYMQLSSLTSEDSAVYYCARSTYYGGDWYFNVWGAGTTVTVSAASTKGPSVFPLAPSSKSTSGGTAALGCLVKDYFPEPVTVSWNSGALTSGVHTFPAVLQSSGLYSLSSVVTVPSSSLGTQTYICNVNHKPSNTKVDKKVEPKSCDKTHTCPPCPAPELLGGPSVFLFPPKPKDTLMISRTPEVTCVVVDVSHEDPEVKFNWYVDGVEVHNAKTKPREEQYNSTYRVVSVLTVLHQDWLNGKEYKCKVSNKALPAPIEKTISKAKGQPREPQVYTLPPSRDELTKNQVSLTCLVKGFYPSDIAVEWESNGQPENNYKTTPPVLDSDGSFFLYSKLTVDKSRWQQGNVFSCSVMHEALHNHYTQKSLSLSPGK	451
8005	alemtuzumab	IgG1k	L	Km3	1	DIQMTQSPSSLSASVGDRVTITCKASQNIDKYLNWYQQKPGKAPKLLIYNTNNLQTGVPSRFSGSGSGTDFTFTISSLQPEDIATYYCLQHISRPRTFGQGTKVEIKRTVAAPSVFIFPPSDEQLKSGTASVVCLLNNFYPREAKVQWKVDNALQSGNSQESVTEQDSKDSTYSLSSTLTLSKADYEKHKVYACEVTHQGLSSPVTKSFNRGEC	214
			H	G1m17,1	4	QVQLQESGPGLVRPSQTLSLTCTVSGFTFTDFYMNWVRQPPGRGLEWIGFIRDKAKGYTTEYNPSVKGRVTMLVDTSKNQFSLRLSSVTAADTAVYYCAREGHTAAPFDYWGQGSLVTVSSASTKGPSVFPLAPSSKSTSGGTAALGCLVKDYFPEPVTVSWNSGALTSGVHTFPAVLQSSGLYSLSSVVTVPSSSLGTQTYICNVNHKPSNTKVDKKVEPKSCDKTHTCPPCPAPELLGGPSVFLFPPKPKDTLMISRTPEVTCVVVDVSHEDPEVKFNWYVDGVEVHNAKTKPREEQYNSTYRVVSVLTVLHQDWLNGKEYKCKVSNKALPAPIEKTISKAKGQPREPQVYTLPPSRDELTKNQVSLTCLVKGFYPSDIAVEWESNGQPENNYKTTPPVLDSDGSFFLYSKLTVDKSRWQQGNVFSCSVMHEALHNHYTQKSLSLSPGK	451
4irz	natalizumab	IgG4k	L†	Km3	1	DIQMTQSPSSLSASVGDRVTITCKTSQDINKYMAWYQQTPGKAPRLLIHYTSALQPGIPSRFSGSGSGRDYTFTISSLQPEDIATYYCLQYDNLWTFGQGTKVEIKRTVAAPSVFIFPPSDEQLKSGTASVVCLLNNFYPREAKVQWKVDNALQSGNSQESVTEQDSKDSTYSLSSTLTLSKADYEKHKVYACEVTHQGLSSPVTKSFNR	210
			H†	-	1	QVQLVQSGAEVKKPGASVKVSCKASGFNIKDTYIHWVRQAPGQRLEWMGRIDPANGYTKYDPKFQGRVTITADTSASTAYMELSSLRSEDEAVYYCAREGYYGNYGVYAMDYWGQGTLVTVSSASTKGPSVFPLAPCSRSTSESTAALGCLVKDYFPEPVTVSWNSGALTSGVHTFPAVLQSSGLYSLSSVVTVPSSSLGTKTYTCNVDHKPSNTKVDKRVESKYGPPCPSCPAPEFLGGPSVFLFPPKPKDTLMISRTPEVTCVVVDVSQEDPEVQFNWYVDGVEVHNAKTKPREEQFNSTYRVVSVLTVLHQDWLNGKEYKCKVSNKGLPSSIEKTISKAKGQPREPQVYTLPPSQEEMTKNQVSLTCLVKGFYPSDIAVEWESNGQPENNYKTTPPVLDSDGSFFLYSRLTVDKSRWQEGNVFSCSVMHEALHNHYTQKSLSLSLGK	450
2H7.v16	ocrelizumab	IgG1k	L	Km3	1	DIQMTQSPSSLSASVGDRVTITCRASSSVSYMHWYQQKPGKAPKPLIYAPSNLASGVPSRFSGSGSGTDFTLTISSLQPEDFATYYCQQWSFNPPTFGQGTKVEIKRTVAAPSVFIFPPSDEQLKSGTASVVCLLNNFYPREAKVQWKVDNALQSGNSQESVTEQDSKDSTYSLSSTLTLSKADYEKHKVYACEVTHQGLSSPVTKSFNRGEC	471
			H	G1	3	EVQLVESGGGLVQPGGSLRLSCAASGYTFTSYNMHWVRQAPGKGLEWVGAIYPGNGDTSYNQKFKGRFTISVDKSKNTLYLQMNSLRAEDTAVYYCARVVYYSNSYWYFDVWGQGTLVTVSSASTKGPSVFPLAPSSKSTSGGTAALGCLVKDYFPEPVTVSWNSGALTSGVHTFPAVLQSSGLYSLSSVVTVPSSSLGTQTYICNVNHKPSNTKVDKKVEPKSCDKTHTCPPCPAPELLGGPSVFLFPPKPKDTLMISRTPEVTCVVVDVSHEDPEVKFNWYVDGVEVHNAKTKPREEQYNSTYRVVSVLTVLHQDWLNGKEYKCKVSNKALPAPIEKTISKAKGQPREPQVYTLPPSREEMTKNQVSLTCLVKGFYPSDIAVEWESNGQPENNYKTTPPVLDSDGSFFLYSKLTVDKSRWQQGNVFSCSVMHEALHNHYTQKSLSLSPGK	232
7860	adalimumab	IgG1k	L†	Km3	1	DIQMTQSPSSLSASVGDRVTITCRASQGIRNYLAWYQQKPGKAPKLLIYAASTLQSGVPSRFSGSGSGTDFTLTISSLQPEDVATYYCQRYNRAPYTFGQGTKVEIKRTVAAPSVFIFPPSDEQLKSGTASVVCLLNNFYPREAKVQWKVDNALQSGNSQESVTEQDSKDSTYSLSSTLTLSKADYEKHKVYACEVTHQGLSSPVTKSFNRGEC	214
			H†	G1m17,3,2	3	EVQLVESGGGLVQPGRSLRLSCAASGFTFDDYAMHWVRQAPGKGLEWVSAITWNSGHIDYADSVEGRFTISRDNAKNSLYLQMNSLRAEDTAVYYCAKVSYLSTASSLDYWGQGTLVTVSSASTKGPSVFPLAPSSKSTSGGTAALGCLVKDYFPEPVTVSWNSGALTSGVHTFPAVLQSSGLYSLSSVVTVPSSSLGTQTYICNVNHKPSNTKVDKKVEPKSCDKTHTCPPCPAPELLGGPSVFLFPPKPKDTLMISRTPEVTCVVVDVSHEDPEVKFNWYVDGVEVHNAKTKPREEQYNSTYRVVSVLTVLHQDWLNGKEYKCKVSNKALPAPIEKTISKAKGQPREPQVYTLPPSRDELTKNQVSLTCLVKGFYPSDIAVEWESNGQPENNYKTTPPVLDSDGSFFLYSKLTVDKSRWQQGNVFSCSVMHEALHNHYTQKSLSLSPGK	451

[1] Identifier in IMGT mAb database or patent filings

[2] Closest (mouse/)human family, determined by IMGT V-quest.

† Imputed constant region sequence based on allotype described in literature.

Figures S1 Artificial neural network perceptron topology

The perceptron used for prediction of cleavage has three layers: an input layer consisting of the vectors of the first three principal components of the amino acids in the octamer binding site; a hidden layer consisting of eight nodes with symmetry to the octamer binding site; and a single output layer, which is the cleavage prediction. A hyperbolic tangent activation function was used for all interconnections within the perceptron structure.

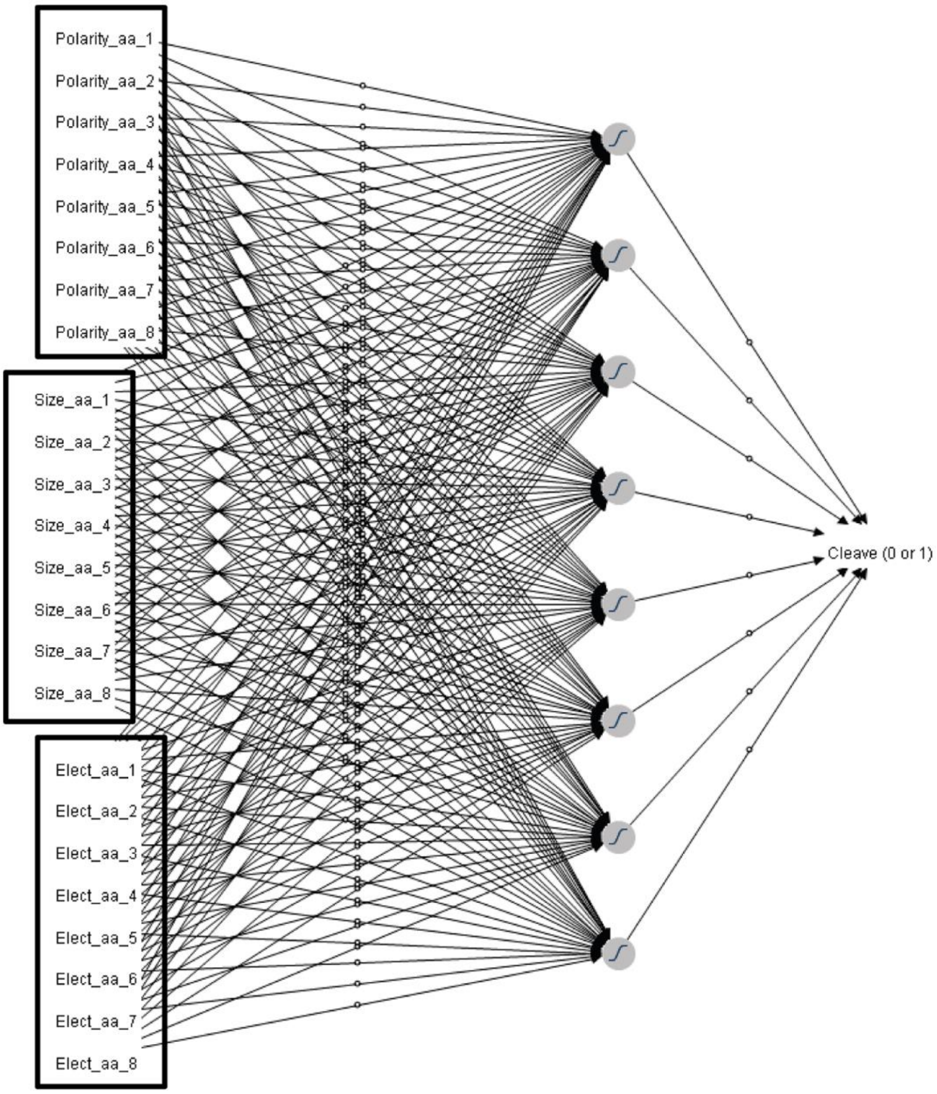

Figure S2 Data flow

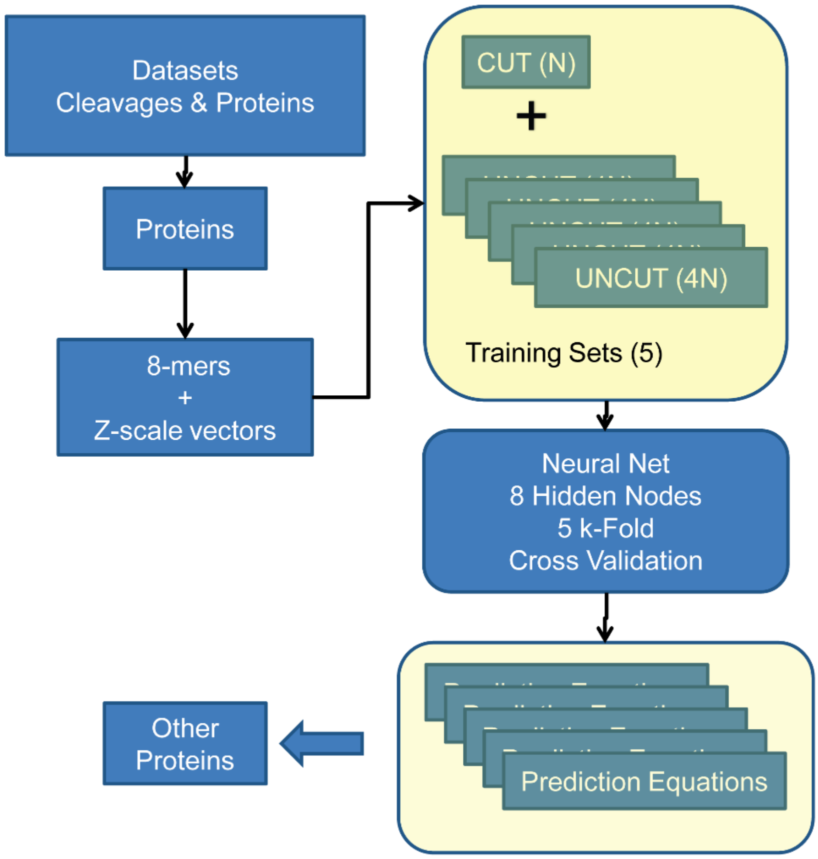

Experimentation showed that a common prediction scheme for all scissile bonds was not achievable with the tools available. Thus, as an alternative a separate neural network prediction ensemble for each scissile bond dipeptide was developed. Of the 400 theoretical dipeptides there were for cathepsin (Cat)S 342, for CatB 272 and for CatL 255 dipeptides in the data set of Biniossek et al (1). The data sets of the cathepsins used had partially overlapping scissile bond preferences and each of the different cathepsins had a subset that were not cleaved. Biniossek et al (1) also indicated a preference for certain amino acids in the P2 position that is also seen in the MEROPS database (https://www.ebi.ac.uk/merops/). Consistent with that observation there were also partially overlapping P2P1 preferences between the different cathepsins. Potential scissile bonds without training sets are coded as missing values (not as zero). It was also found that the same scissile bond dipeptide occurred in many more uncleaved CSO than in those that were cleaved. Thus, a bagging process was developed where random training sets were assembled for each dipeptide and each of the training sets contained 5 times as many uncleaved CSO as cleaved CSO. These training sets were used in a 5k-Fold cross validation. The following pseudocode outlines the basic data assembly and data processing activity that were used to collate the training cleaved peptides

and their cohorts from the same protein set but that were not cleaved. This results in this article were derived with a probabilistic neural network and used a 5-kfold process cross validation process, others could be used. We have also used the process successfully with recursive partitioning and support vector machines.

Bootstrap Aggregating ("bagging") Training Set Assembly
Input:
1. Cleaved octomers derived from proteomic cleavage data sets
2. Uncleaved octomers from same proteins derived from octomer-windowing the protein sequences by single amino acid displacement of intact proteins in the proteomic cleavage data sets.

Output:
1. Training sets of matched ratios of cleaved:uncleaved octomers for each amino acid (A,C,D,E,F,G,H,I,K,L,M,N,P,Q,R,S,T,V,W,Y) found at the P1 position of the CSO (<= 20)
2. Training sets of matched ratios of cleaved:uncleaved octomers for each amino acid (A,C,D,E,F,G,H,I,K,L,M,N,P,Q,R,S,T,V,W,Y) found at the P1' position of the CSO (<= 20)
3. Training sets of matched ratios of cleaved:uncleaved octomers sets for all P1-P1' combinations (AA … YY) (<= 400)

Preliminary:
1. Create a dataset of singleton octomers from the cleaved and uncleaved data sets from the protein datasets downloaded from the repository.
2. Remove the cleaved octomers from the total set (the downloaded sets of intact proteins will also contain the cleaved octomers as well and they must be removed).

```
Repeat
For Each Ensemble Training Cohort (ETC)
        Process 1:
        For Each P1 anchor amino acid (A,C,D,E,F,G,H,I,K,L,M,N,P,Q,R,S,T,V,W,Y)
                For each cleaved octomer with matching P1
                        Select 4 non-cleaved octomers with matching P1 at random =>) ETC_P1(A … Y)
                End For
        End For
        Process 2:
        For Each P1' anchor amino acid (A,C,D,E,F,G,H,I,K,L,M,N,P,Q,R,S,T,V,W,Y)
                For each cleaved octomer with matching P1'
                        Select 4 non-cleaved octomers with matching P1 at random => ETC_P1'(A … Y)
                End For
        End For
        Process 3:
        For Each P1-P1' dipeptide (A,C,D,E,F,G,H,I,K,L,M,N,P,Q,R,S,T,V,W,Y)as combinatorial pairs
                For each cleaved octomer with matching P1-P1'
                Select 4 non-cleaved octomers with matching P1-P1' at random => ETC_P1-P1'(AA … YY)
                End For
        End For
End For
Until (ETC == 5)
```

Bootstrap Aggregating ("bagging") Classifier Construction
Input:
Multiple ***ETC*** (*e.g.* 5 per (A,C,D,E,F,G,H,I,K,L,M,N,P,Q,R,S,T,V,W,Y) for P1 and P1')
Output:
Multiple (*e.g.* 5) ensembles from each of 5 *ETC* = 25 total classifiers for each P1, P1' and P1-P1'

Note: This process results in a total 50 discriminant equations for each potential scissile bond (25 for the P1 and 25 for the P1' side). As training is done independently concordant predictions of cleavage probability provide added confidence in the results for any particular scissile bond. The average probability of the 25 member equation ensembles is used as the prediction metric.

```
Repeat
        Repeat with each ETC
                Build classifier* discriminant equations using 5-kfold cross validation each from a different
                random starting point
        Until(N repeats == 5)
```

Until (N Classifiers per ***ETC*** == 5)

Classifier use for prediction of cleavage probability within protein sequences
For each sequential pair of amino acids in a protein sequence

1. Compute average probabilities using 25 equation ensembles from the P1 side of the scissile bond that match the particular amino acid pair
2. Compute average probabilities using 25 equation ensembles from the P1’ side of the scissile bond that match the particular amino acid pair

Optionally (if 1 and 2 are discordant):

3. Compute average probabilities using 25 equation ensembles for the P1-P1’ dipeptide pair

End For

Figure S3 Comparing neural net to support vector machine

Comparison of the performance of a probabilistic neural network (NN) and a support vector machine (SVM) as binary classifiers for predicting cleavage of human cathepsin L. The cleavage site octamers in the peptide training sets had either an alanine or a glycine at position P1 (a) and (c) Glycine at P1 Total of the cleaved trainer peptides was 222 (indicated by the blue horizontal line). Cleaved peptides were paired for training with 5 un-cleaved random cohorts with 888 peptides in each set (indicated by red horizontal line). (b) and (d) alanine at P1. Total of the cleaved trainer peptides was 111 (blue horizontal line). Cleaved peptides were paired for training with 5 un-cleaved random cohorts with 444 peptides in each set (red horizontal line).

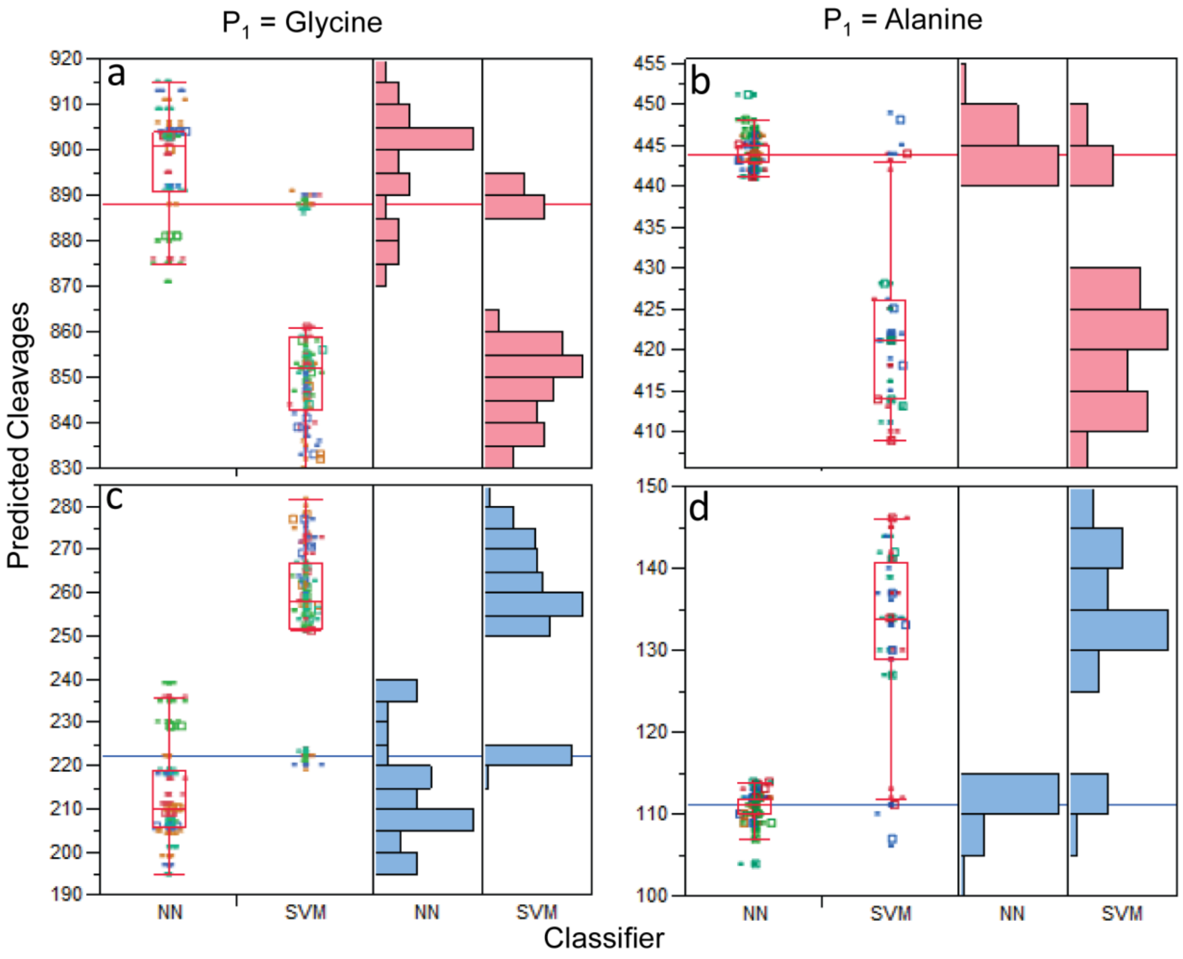

Figure S4 Hierarchical cluster of peptide occurrences by sample

Alpha-synuclein (aSyn), recombinant myelin basic protein (rMBP) isoforms 2 and 6, and tau digested by cathepsin S, L or B at pH 6 for 6, 24 or 30 hours were analyzed using LC-MS to identify resulting peptides. The occurrences of the peptides were compared using pairwise hierarchical clustering of standardized values, by method of Ward.

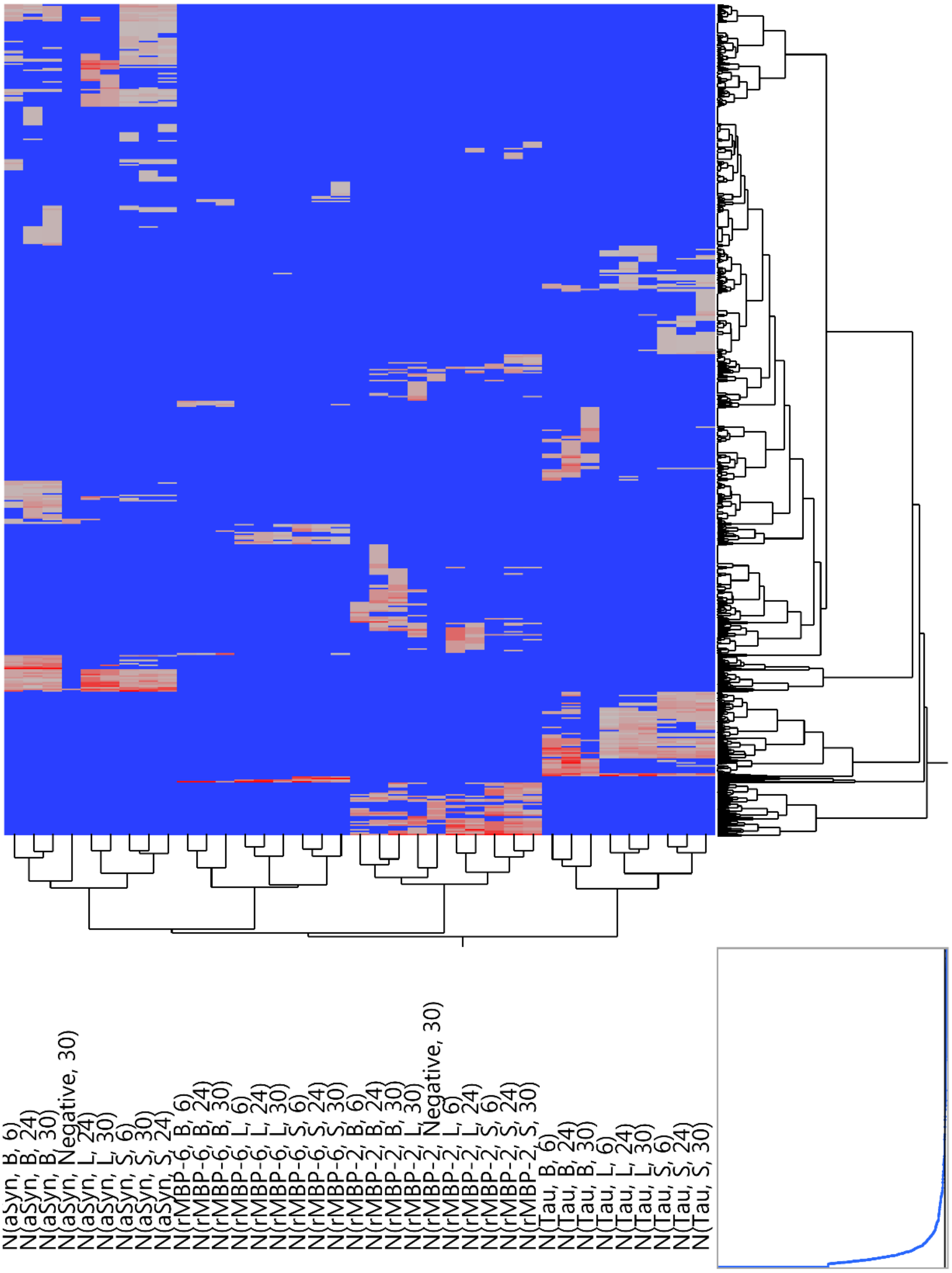

Figure S5 Possible carboxypeptidase activity for cathepsin B

The maximum distance from a high-probability cleavage site (p>0.79) intra protein (rMBP-2) was plotted (X-axis) against the observed number cleavages at the given positions. Each point indicates number of cleavages associated with a given distance, and the lines are smoothed averages.

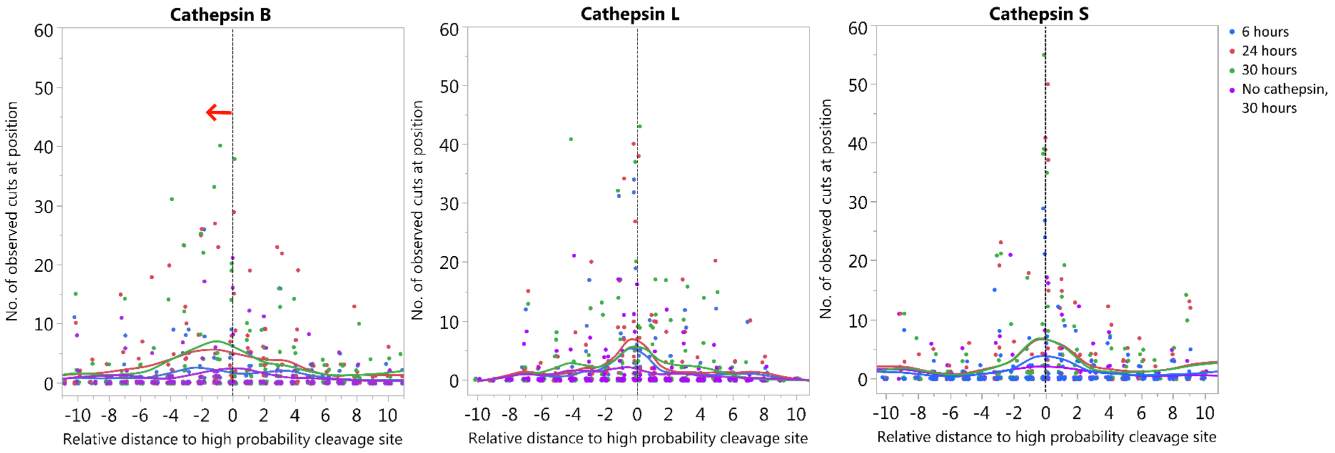

Figure S6 Evaluation of cleavage accuracy for immunoglobulin variable regions

Cleavage probability for all possible cleavage site octamers (CSOs) within A) heavy and B) light chain variable regions of rituximab, infliximab, ocrelizumab, natalizumab, alemtuzumab and adalimumab were binned into ranges of 0.2 (X-axis). Intra-chain z-standardized number of observed cuts after 24 hours at pH 6 are depicted on the Y-axis. P-values indicate Welch ANOVA significance, and differing letters indicate significant differences between groups (Tukey-Kramer, HSD).

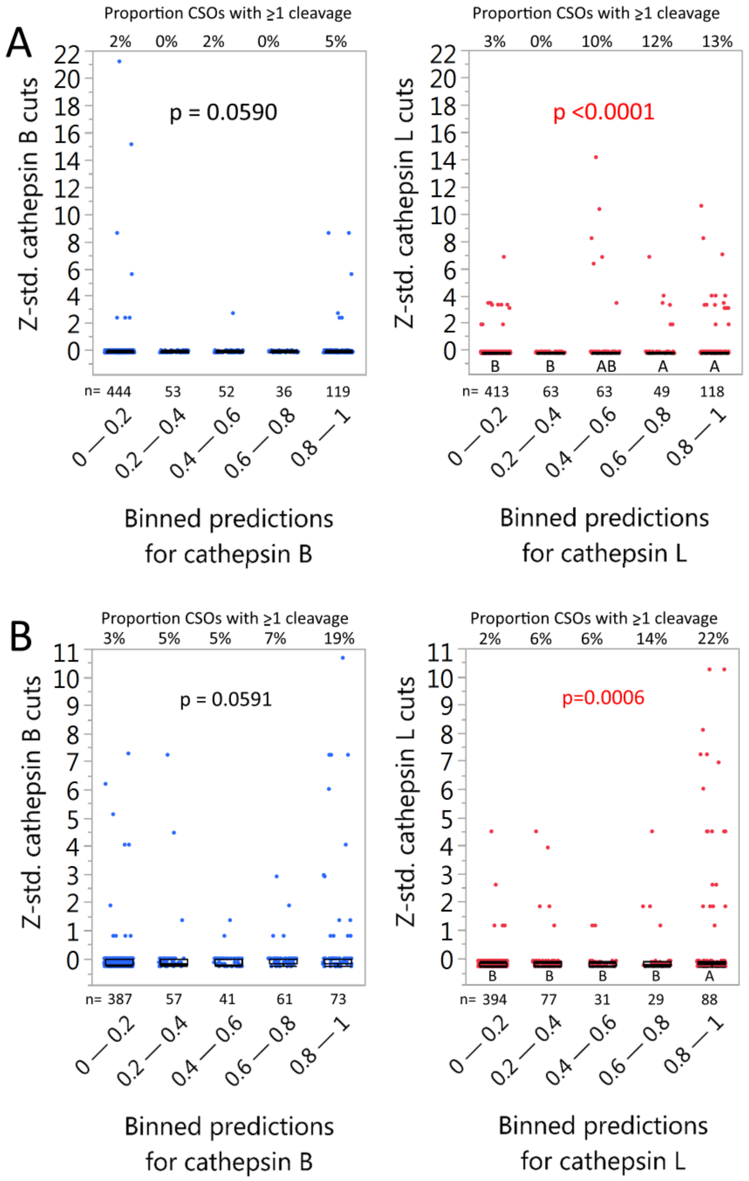

Figure S7 Adalimumab digestion pattern at different pH

Non-standardized number of observed cuts with LC/MS within the Ig light chain after incubating adalimumab with a) cathepsin S, b) cathepsin L and c) cathepsins B for 24 hours at pHs 4, 5 or 6. For alignment purposes, the position is assigned by the relative position of P1' in the cleavage site octamer to the cysteine (0) of CDR3. The constant region is aligned to start at position 30.

A)

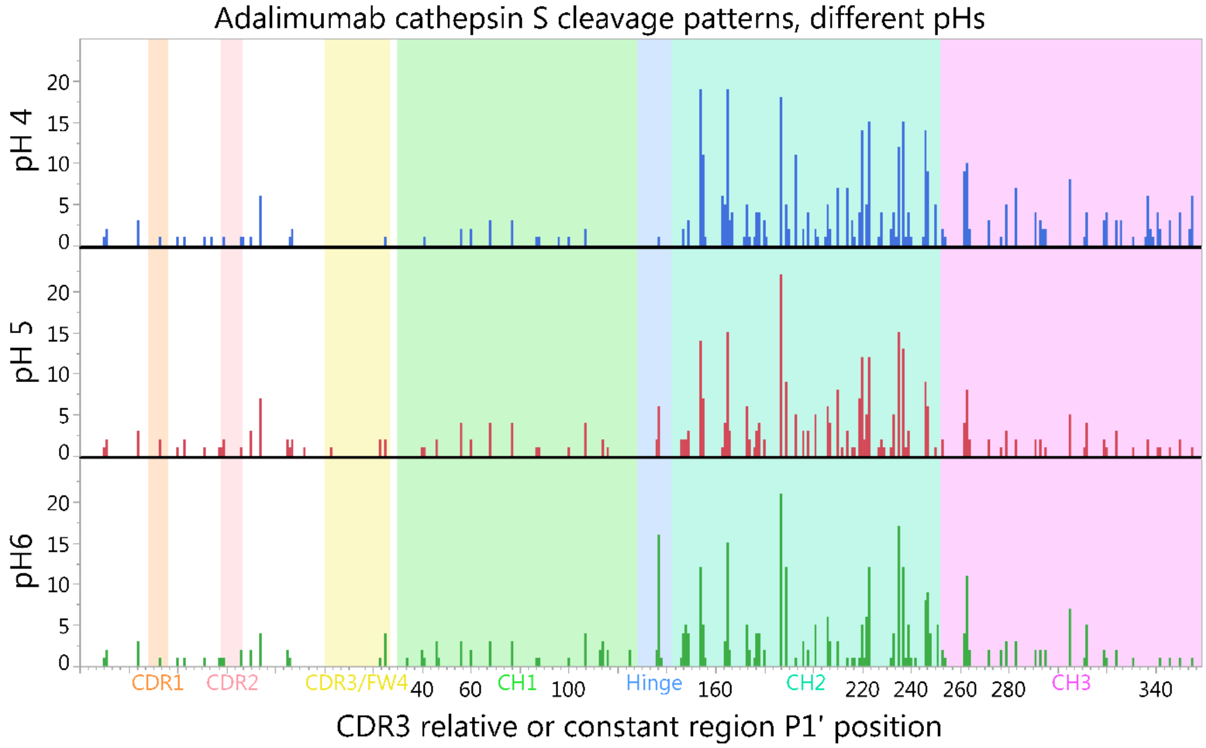

B)

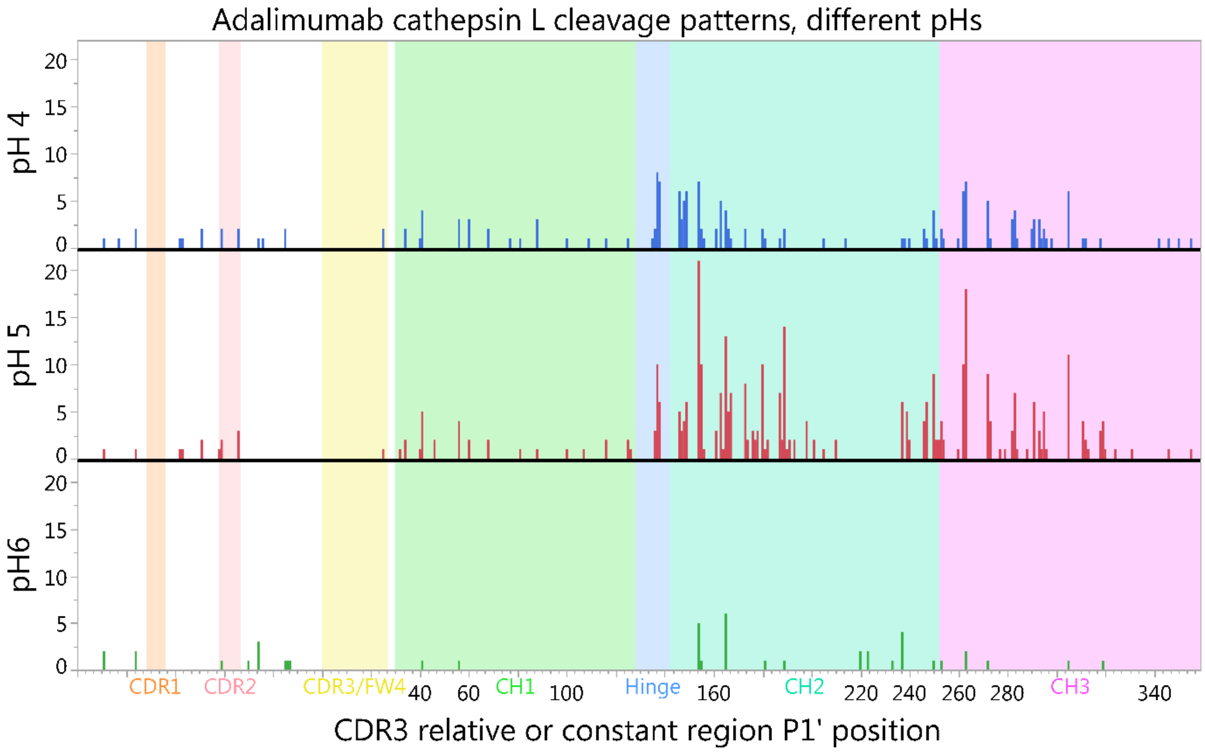

Adalimumab cathepsin L cleavage patterns, different pHs
pH 4
pH 5
pH6
20
15
10
5
0
CDR1
CDR2
CDR3/FW4
40
60
CH1
100
Hinge
160
CH2
220
240
260
280
CH3
340
CDR3 relative or constant region P1' position

C)

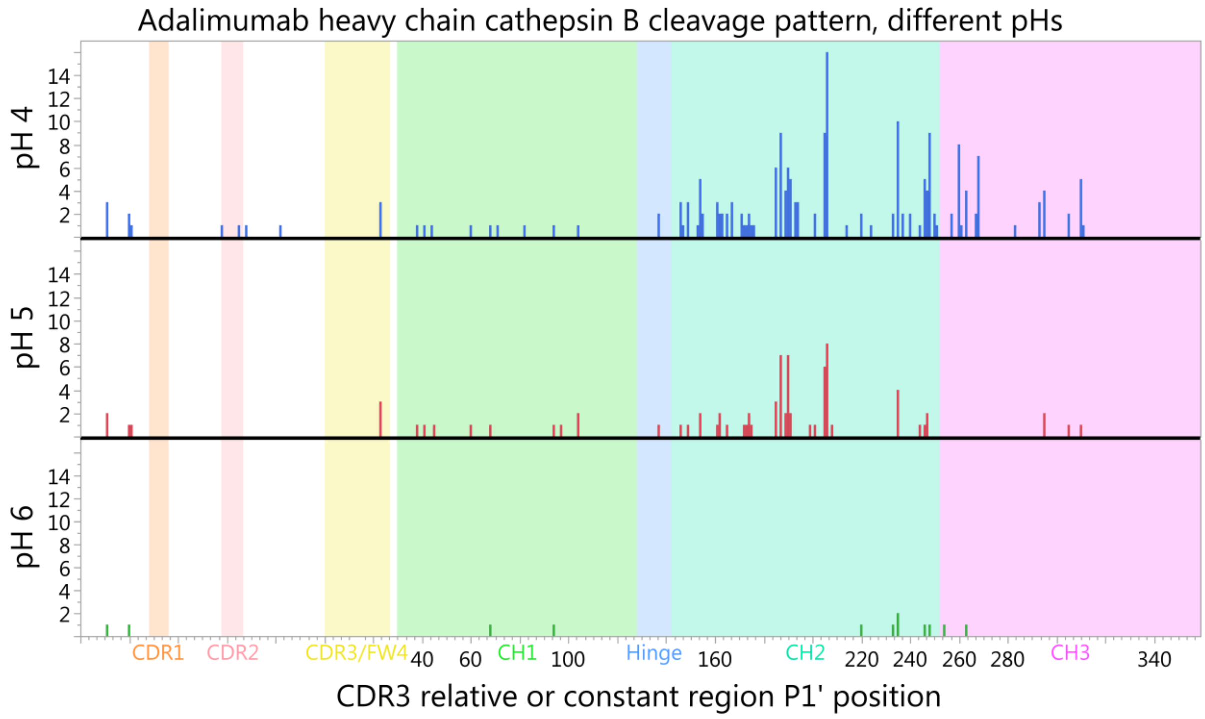

Adalimumab heavy chain cathepsin B cleavage pattern, different pHs
pH 4
pH 5
pH 6
14
12
10
8
6
4
2
CDR1
CDR2
CDR3/FW4
40
60
CH1
100
Hinge
160
CH2
220
240
260
280
CH3
340
CDR3 relative or constant region P1' position

Figure S8 Infliximab digestion by cathepsin S, L and B at pH 4, 5 and 6.

Distribution of peptide lengths after digestion of 2400 nM infliximab with either cathepsin B, L, S at 6, 24 or 30 hours at pH 4, 5 or 6. Each datapoint represents one identified peptide at the given time point. Black lines with annotations indicate the mean size of peptides. Purple and green areas indicate peptide sizes fitting HLA class I and II, respectively.

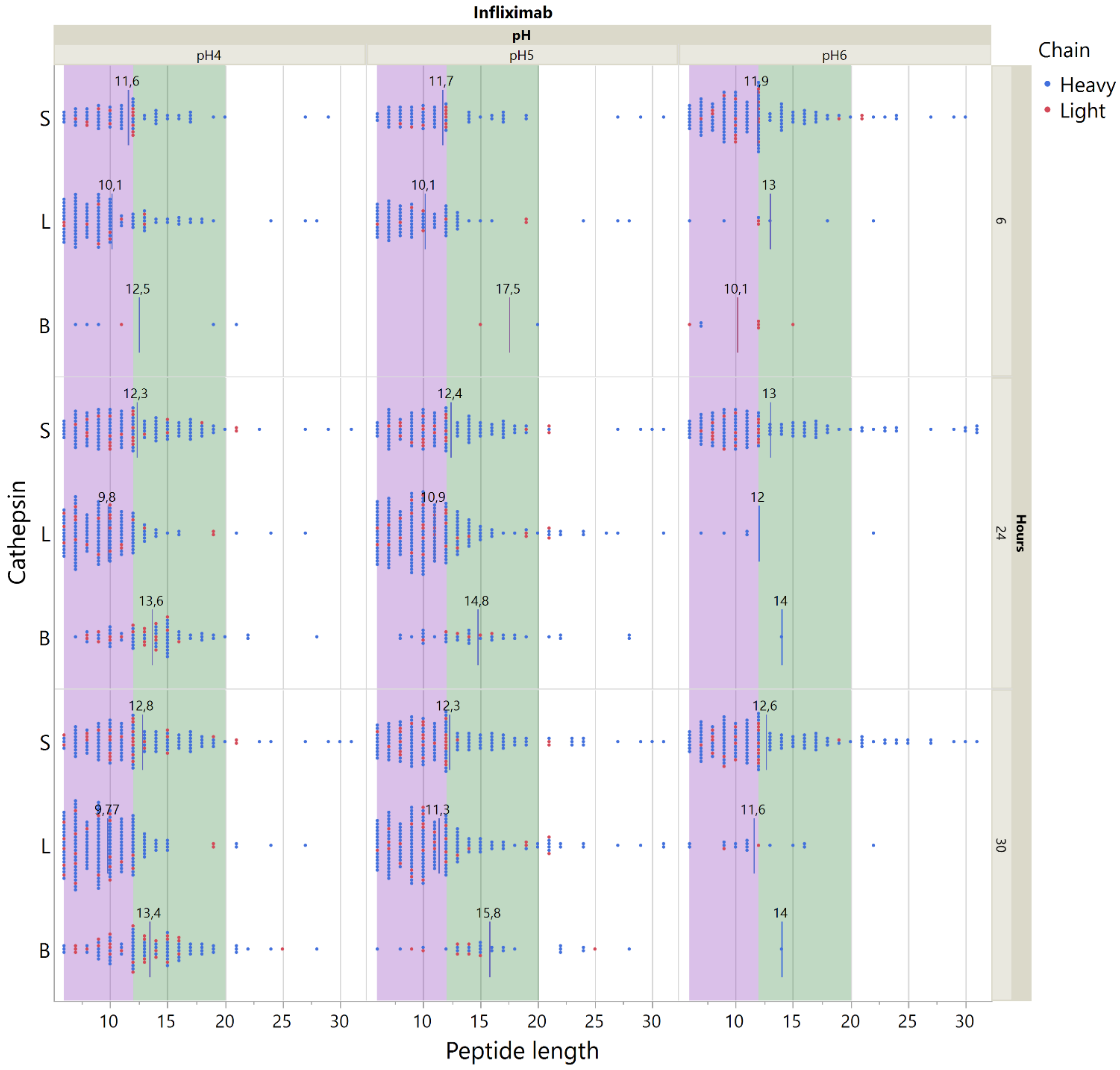

Figure S9 SDS-PAGE of digested IgGs

One µg of adalimumab (A) or ocrelizumab (B) digested by cathepsins S, L or B for 2 or 30 hours in presence 4 mM DTT of per well, were stained with Coomassie blue. Adalimumab and ocrelizumab samples incubated with 4 mM DTT alone (Neg) and unmanipulated batch samples were used as controls. Findings in lane 11 (Cathepsin L, pH 4, 30 hours) were replicated using both adalimumab and ocrelizumab samples (not shown). A weaker band with the same location was also found with cathepsin L at pH 5 (Figure S8B).

A)

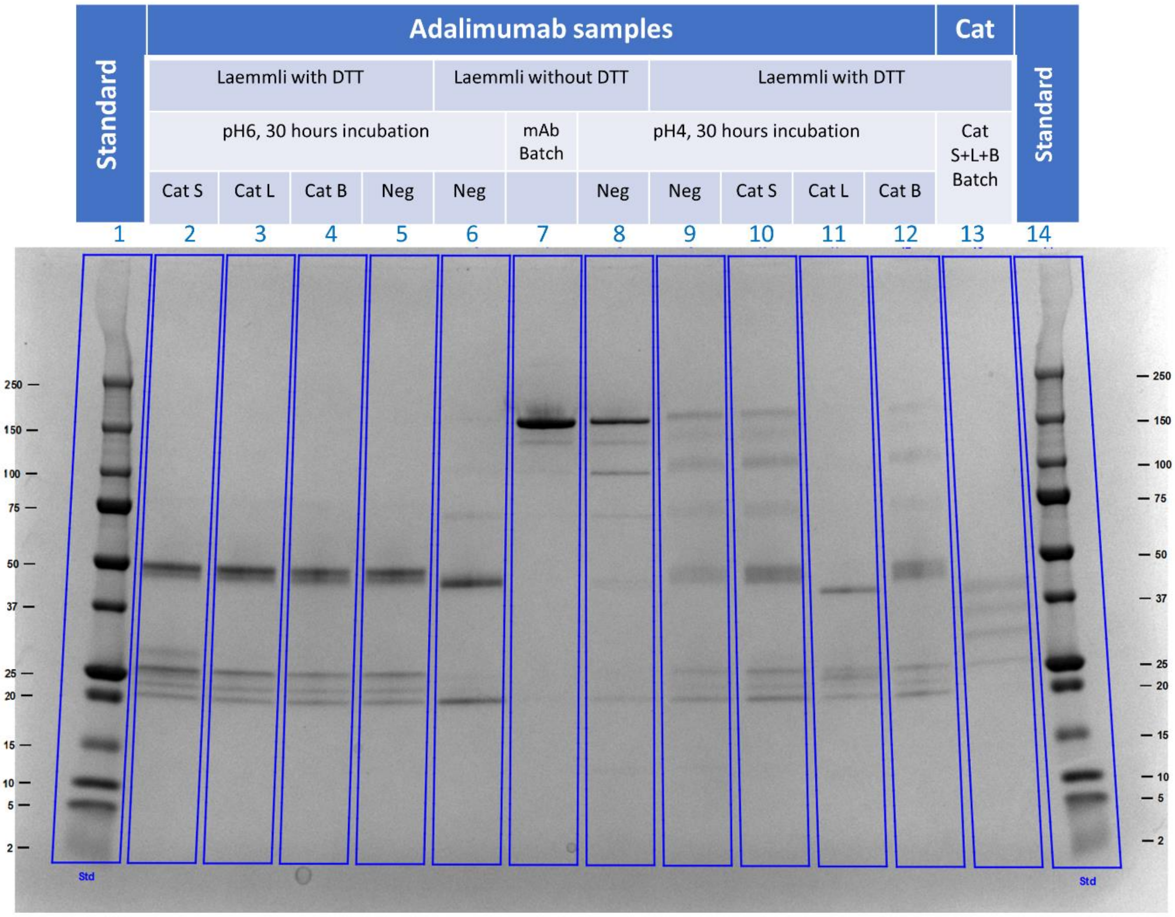

B)

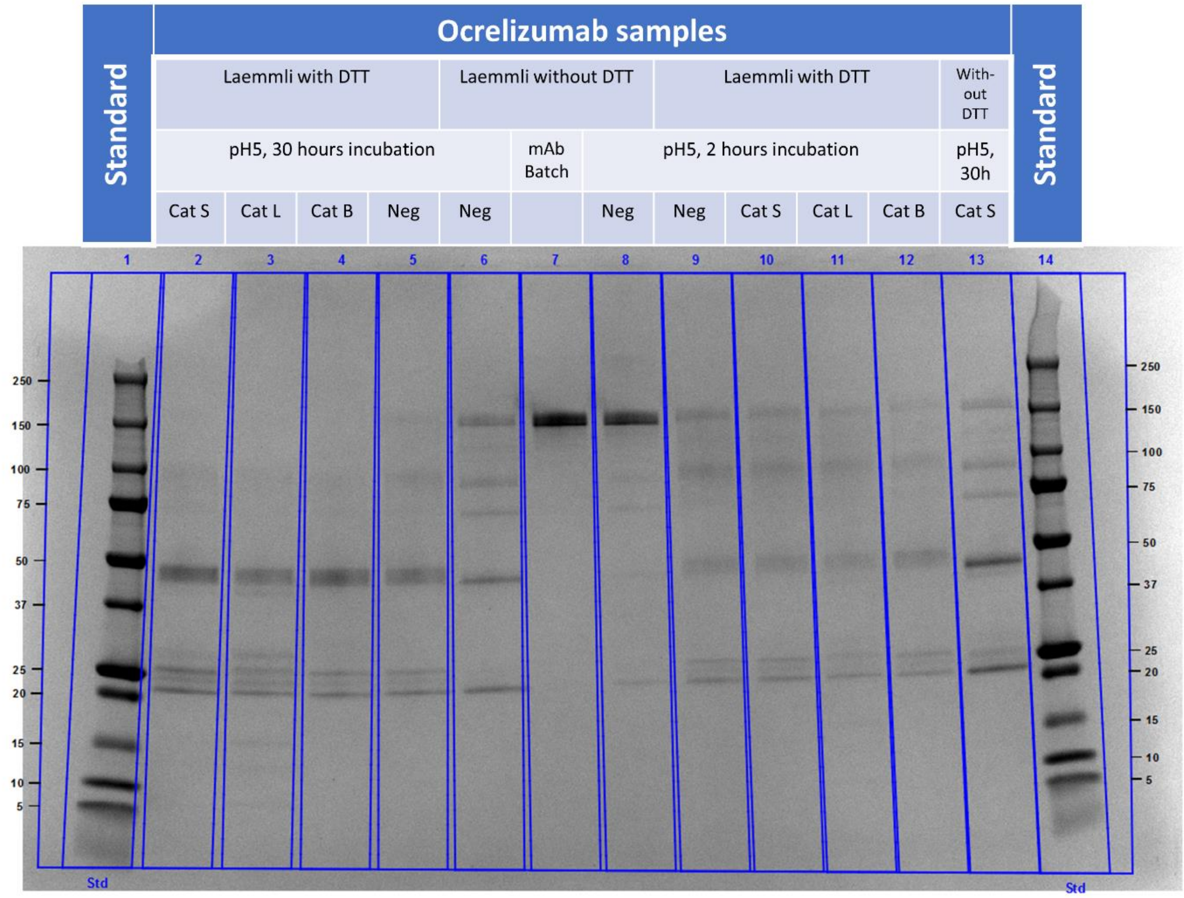

Standard
Ocrelizumab samples
Laemmli with DTT
Laemmli without DTT
Laemmli with DTT
With-out DTT
pH5, 30 hours incubation
mAb Batch
pH5, 2 hours incubation
pH5, 30h
Cat S
Cat L
Cat B
Neg
Neg
Neg
Neg
Cat S
Cat L
Cat B
Cat S
Standard
1
2
3
4
5
6
7
8
9
10
11
12
13
14
250
150
100
75
50
37
25
20
15
10
5
Std
250
150
100
75
50
37
25
20
15
10
5
Std

References

1. Biniossek ML, Nagler DK, Becker-Pauly C, Schilling O. Proteomic identification of protease cleavage sites characterizes prime and non-prime specificity of cysteine cathepsins B, L, and S. *Journal of proteome research* (2011) **10**(12):5363-73. Epub 2011/10/05. doi: 10.1021/pr200621z. PubMed PMID: 21967108.
